# Molecular identification of late and terminal Pleistocene *Equus ovodovi* from northeastern China

**DOI:** 10.1371/journal.pone.0216883

**Published:** 2019-05-16

**Authors:** Jun-Xia Yuan, Xin-Dong Hou, Axel Barlow, Michaela Preick, Ulrike H. Taron, Federica Alberti, Nikolas Basler, Tao Deng, Xu-Long Lai, Michael Hofreiter, Gui-Lian Sheng

**Affiliations:** 1 Faculty of Materials Science and Chemistry, China University of Geosciences, Wuhan, China; 2 State Key Laboratory of Biogeology and Environmental Geology, China University of Geosciences, Wuhan, China; 3 Institute for Biochemistry and Biology, University of Potsdam, Potsdam, Germany; 4 Key Laboratory of Vertebrate Evolution and Human Origins of Chinese Academy of Sciences, IVPP, Beijing, China; Universita degli Studi di Pavia, ITALY

## Abstract

The extant diversity of horses (family Equidae) represents a small fraction of that occurring over their evolutionary history. One such lost lineage is the subgenus *Sussemionus*, which is thought to have become extinct during the Middle Pleistocene. However, recent molecular studies and morphological analysis have revealed that one of their representatives, *E*. *ovodovi*, did exist in Siberia during the Late Pleistocene. Fossil materials of *E*. *ovodovi* have thus far only been found in Russia. In this study, we extracted DNA from three equid fossil specimens excavated from northeastern China dated at 12,770–12,596, 29,525–28,887 and 40,201–38,848 cal. yBP, respectively, and retrieved three near-complete mitochondrial genomes from the specimens. Phylogenetic analyses cluster the Chinese haplotypes together with previously published Russian *E*. *ovodovi*, strongly supporting the assignment of these samples to this taxon. The molecular identification of *E*. *ovodovi* in northeastern China extends the known geographical range of this fossil species by several thousand kilometers to the east. The estimated coalescence time of all *E*. *ovodovi* haplotypes is approximately 199 Kya, with the Chinese haplotypes coalescing approximately 130 Kya. With a radiocarbon age of 12,770–12,596 cal. yBP, the youngest sample in this study represents the first *E*. *ovodovi* sample dating to the terminal Pleistocene, moving the extinction date of this species forwards considerably compared to previously documented fossils. Overall, comparison of our three mitochondrial genomes with the two published ones suggests a genetic diversity similar to several extant species of the genus *Equus*.

## Introduction

*Sussemionus* is a recently described subgenus of *Equus* that is thought to have originated in Alaska during the Pliocene. Its representatives were widely distributed from North America to Eurasia and Africa [1, 2]. Although fossil materials of *Sussemionus* are abundant, its evolutionary history is not yet well understood. It was previously believed that all its members were restricted to the Early and Middle Pleistocene [1]. However, ancient DNA analyses and fossil evidence from Proskuriakova cave and Denisova cave in Russia verified that at least one species of *Sussemionus*, *E*. *ovodovi*, survived as recently as the Late Pleistocene [1, 3–6].

*E*. *ovodovi*, was a confusing fossil species in terms of its morphological features. Specimens excavated in Proskuriakova cave (Khakassia, southeastern part of Western Siberia, Russia) were previously identified as *Equus hydruntinus* based on morphological characteristics, while later genetic studies revealed that they instead formed a separate lineage in the *Equus* phylogenetic tree [3]. Further morphological studies suggested that these specimens are a member of the subgenus *Sussemionus*, which were subsequently named as a different species, *E*. *ovodovi* [1, 2]. These specimens were dated to the Late Pleistocene according to the stratigraphic layer, which challenged the previous view of *Sussemionus* having gone extinct approximately half a million years ago [2, 4]. Following this discovery, paleontologists identified some additional Late Pleistocene *E*. *ovodovi* fossils from Khakassia, in the Altai Mountains, and alluvial deposits in the Altai piedmont lowlands in Russia [2, 4, 7]. At present, however, no Late Pleistocene *E*. *ovodovi* specimens have been reported from outside the political boundaries of present-day Russia.

Besides palaeontological and morphological studies, evolutionary biologists have carried out further exploration on *E*. *ovodovi* at the molecular level. Complete mitochondrial genomes were retrieved from two Late Pleistocene *E*. *ovodovi* specimens collected from Proskuriakova cave and Denisova cave, respectively [5, 6]. The mitochondrial genomes were used to investigate the phylogenetic relationships of *E*. *ovodovi* and other extant and extinct equids. Vilstrup *et al*. [5] suggested that *E*. *ovodovi* formed the sister clade of all sampled non-caballine equids, which was similar to previous results [3] using shorter mitochondrial DNA sequences. However, a recent study suggested that *E*. *ovodovi* shared more recent common ancestry with the zebra clade than with *Equus asinus*, *E*. *hemionus* or *Equus kiang* [6]. The above mentioned studies, however, did not provide notable statistical support for the phylogenetic position of *E*. *ovodovi*.

Numerous equid fossils have been found in Pleistocene strata in Northern China [8–10]. Paleontologists have carried out detailed morphological studies on these fossils [9, 11–12], concluding that *E*. *hemionus*, *Equus przewalskii*, and the extinct *Equus dalianensis* are present in Late Pleistocene deposits in Northern China [9, 11, 13–15]. In this study, we analyzed equid fossil specimens excavated from northeastern China and demonstrated the existence of a fourth horse species in Late Pleistocene China, *E*. *ovodovi*, representing the first documented occurrence of this taxon outside of present day Russia.

## Materials and methods

### Samples

**Fig 1:**
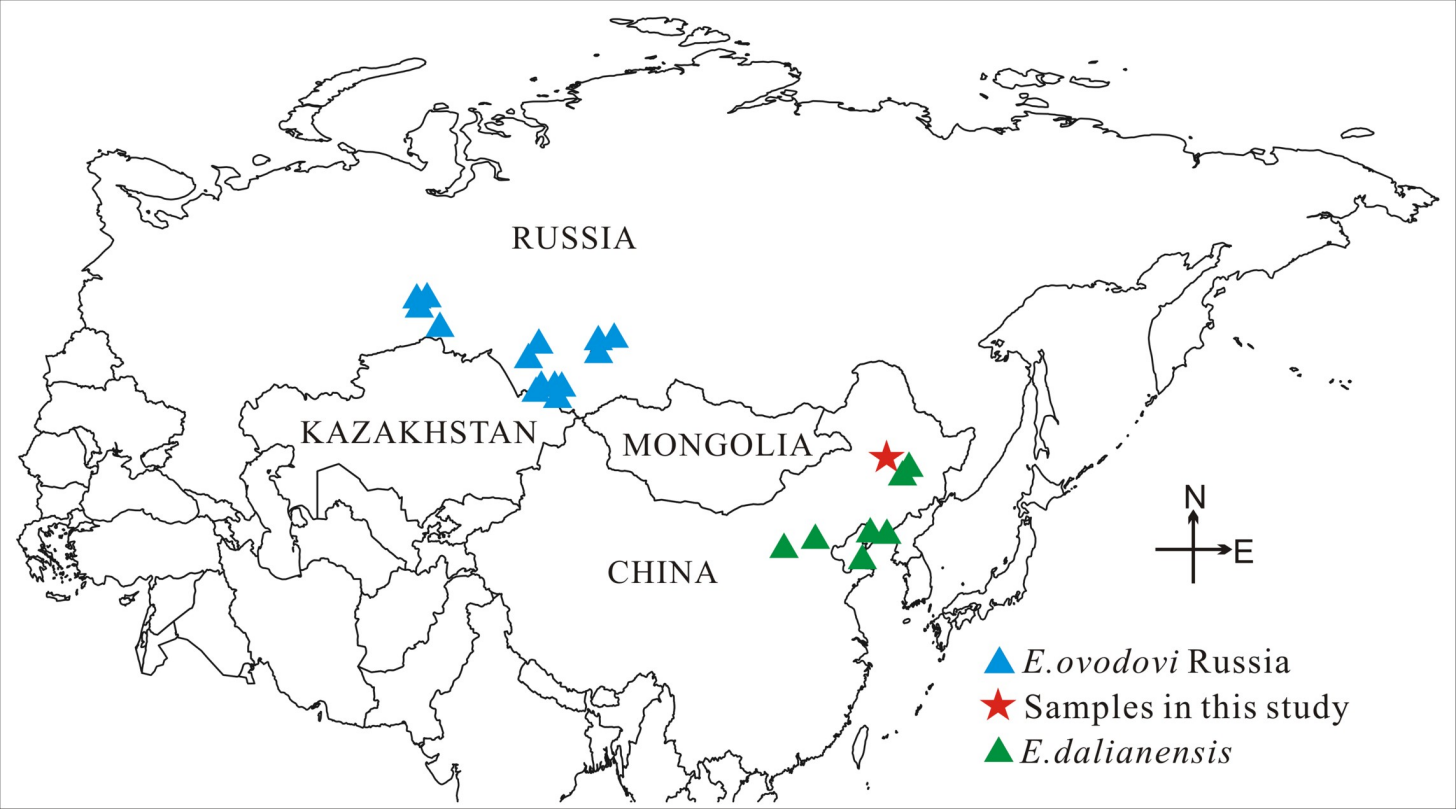
Location of sampling sites in this study and fossil findings of *E*. *ovodovi* and *E*. *dalianensis*. Sampling site in this study is indicated by a red star; the *E*. *ovodovi* specimens in Russia are shown by blue triangles; fossil findings of *E*. *dalianensis* according to the literature are shown by green triangles.

One third metacarpal bone and two third metatarsal bones of equids were excavated from Kunni River bed at Taiping village, Zhaodong county, Heilongjiang Province, northeastern China (Fig 1). The excavation was carried out by a local farmer within the non-written permission from the local government, which includes the undertaking of dredging the Kunni River and collecting deposits in the river bed. The samples were then handed in to Daqing Museum for a scientific analysis and transferred to the ancient DNA group at China University of Geosciences (Wuhan) for molecular analysis. Three specimens in this study (ZDT4, ZDT7, and ZDT9) were AMS-radiocarbon dated at the Archaeological Geochronology Laboratory of Peking University (PKUAMS). Calibration was done using IntCal13 [16], and yielded ages of 12,770–12,596 (ZDT9), 29,525–28,887 (ZDT7) and 40,201–38,848 (ZDT4) cal. yBP (BA170550-52, PKUAMS, China), respectively. The remaining of all samples were returned to Daqing Museum after all molecular experiments and phylogenetic analyses were conclued. They were numbered as H52707 (ZDT4), H52708 (ZDT7), and H52709 (ZDT9) and will remain publicly accessible and available to researchers in the field.

### DNA extraction and library construction

Ancient DNA extraction and sequencing library construction were carried out in a dedicated ancient DNA laboratory. Total genomic DNA was extracted from the specimens according to the method described by Dabney *et al*. [17], while adding 650 μL rather than 750 μL PE buffer at the membrane washing step, and carrying out centrifugation steps at 1,500 rpm for 4 min. In addition, for every four samples, one blank control was added when performing DNA extraction.

A total volume of 20 μL DNA extract of each sample was used for the preparation of sequencing libraries. Single-stranded DNA libraries were prepared following Gansauge & Meyer [18]. The enzymes Uracil-DNA glycosylase and Endonuclease VIII were used to excise uracil residues likely to result from cytosine deamination, and cleave the resulting abasic sites. We also reduced the amount of Circligase II to 2 μL (100 U/μL) in the ligation step of the first adapter, and increased the incubation time to overnight at 60°C [19]. The optimal number of cycles for dual-indexing library amplification was estimated in advance using qPCR [18–19]. Blank controls were additionally included during library preparation to monitor for potential contamination.

### Hybridization capture

The sequencing libraries were enriched for mitochondrial sequences by hybridization capture as described in Maricic *et al*. [20]. DNA extraction and library blank controls were also subjected to the same hybridization capture procedure. First, we prepared the library baits: a modern horse complete mitochondrial genome was amplified from DNA extracted from commercial pet food using long range polymerase chain reaction (LR-PCR). Four LR-PCR primer pairs from a previous study [5] were used with minor modification (S1 Table). The amplified modern horse mitochondrial DNA sequences were sheared to approximately 200 bp fragments using the Covaris S220 ultrasonicator; then the sheared DNA fragments were blunt-end repaired and ligated to biotinylated adapters.

In order to improve the enrichment, hybridization capture was carried out for two rounds [20]. Firstly, we pooled the library and bait in a ratio of 10:1 at a final volume of 18.6 μL for each sample, and added 41.4 μL hybridization buffer mixture to each library-bait pool. Secondly, we incubated the hybridization reactions in a thermocycler at 95°C for 5 min, cooled down to 65°C at 0.1°C/sec, and then incubated hybridization reactions at 65°C for 24 h. We then added 6 μL magnetic beads to each hybridization reaction, immobilized the libraries on the beads under constant rotation in the oven at 22°C for 20 min, and washed the beads with BWT buffer, HW buffer and TET buffer. Lastly, we added 30 μL of TET buffer to the pelleted beads and incubated the pelleted beads at 95°C for 5 min, to elute the enriched libraries from the beads. Subsequently, we continued with post-capture amplification of the enriched libraries.

Next, the amplified libraries were purified using commercial silica spin columns (Qiagen MinElute) and quantified by using a TapeStation Instrument (Agilent). The captured libraries were sequenced on the Illumina Nextseq 500 platform generating 75bp single-end reads following the procedures described in Paijmans *et al*. [21].

### Data analysis

First, Illumina reads were trimmed to remove adapters by using Cutadapt v1.4.2 [22] and reads shorter than 30 bp were discarded. The trimmed reads were aligned to several different reference mitochondrial genomes: *E*. *asin*us (GenBank: X97337), *Equus caball*us (GenBank: X79547), and *E*. *ovodo*vi (GenBank: KY114520), using the “aln” algorithm with default parameters in the Burrows-Wheeler aligner (BWA) [23]. Sequences with a map quality score less than 30 were excluded by samtools v0.1.19-44428cd [24] “view” and then the alignment was sorted by mapping position using “sort”. Sequences with identical 5’ mapping coordinates were collapsed by “rmdup”. Finally, a consensus sequence based on maximum effective read depth was generated for each alignment by using ANGSD v0.916 [25].

To investigate the phylogenetic relationships of these three haplotypes in relation to published equid sequences, we computed a maximum-likelihood tree in RAxML-HPC2 v.8.2.3 [26] using a data set of the complete mitochondrial genome sequences obtained in this study together with the published sequences of *Equus* specimens available from GenBank. We produced a multiple genome alignment of 118 equids and six *Hippidion* complete mtDNA sequences using MUSCLE v.3.8.31 [27]. The maximum-likelihood tree was calculated under the GTR+GAMMA model and the statistical support for clade assessed using 1,000 bootstrap replicates under the GTR+CAT model, with the *Hippodion* clades specified as outgroup to root the tree.

We estimated the timings of coalescence of the five available *E*. *ovodovi* haplotypes using Bayesian inference in BEAST v.1.8.2 [28]. The five sequences were aligned using MUSCLE v.3.8.31 producing 16,445 aligned nucleotide positions and 213 variable sites. The small number of sequences and low variability prevented the use of very complex phylogenetic models. As such, coalescence times were estimated assuming a constant population size over the time-span of the phylogeny, a strict molecular clock and the HKY+G model of nucleotide substitution. The phylogeny was calibrated by tip dating, using median calibrated radiocarbon ages for all sequences [6] except the Russian sequence JX312734, for which an age of 40,000 years was applied based on stratigraphic evidence [5]. The substitution rate was estimated within an open, uniform prior of 0–20% per million years. The MCMC chain ran for sufficient length to achieve convergence and adequate sampling of all parameters (effective sample sizes > 200), verified using TRACER. The maximum clade credibility tree was selected from the posterior sample and annotated with relevant statistics using TREEANNOTATOR and viewed in FIGTREE. The BEAST input xml file is included as supplementary information (S1 File).

In addition, we also investigated the variable nucleotide positions of *E*. *ovodovi* based on pairwise distances among the five mitochondrial genomes obtained in this study (ZDT4, ZDT7 and ZDT9) and retrieved from GenBank (GenBank No: KY114520 and JX312734), respectively. We compared the average pairwise distance among *E*. *ovodovi* haplotypes with other non-caballine equids *Equus burchellii*, *Equus grevyi*, *Equus zebra*, *E*. *kiang*, *E*. *hemionus* and *E*. *asinus* (S2 Table). Calculation of Tajima’s Neutrality Test π-value was carried out in MEGA 7 [29], codon positions included were 1st+2nd+3rd+Noncoding, and all positions containing gaps and missing data were eliminated.

## Results

Mapping to an *E*. *ovodovi* reference mitochondrial genome (GenBank No: KY114520) produced complete coverage of the reference with a mean depth of 126.8, 60.8 and 58.1 fold for the three fossil samples, respectively. In contrast, the same trimmed reads covered only 94.2%-91.5% of the *E*. *asinus* reference mitochondrial genome (GenBank No: X97337) with a mean depth of 80.0–33.3 fold. As to *E*. *caballus* (GenBank No: X79547), the trimmed reads only mapped to 90.9%-85.8% of this reference genome with a mean depth 71.1–29.0 fold (S3 Table).

**Fig 2:**
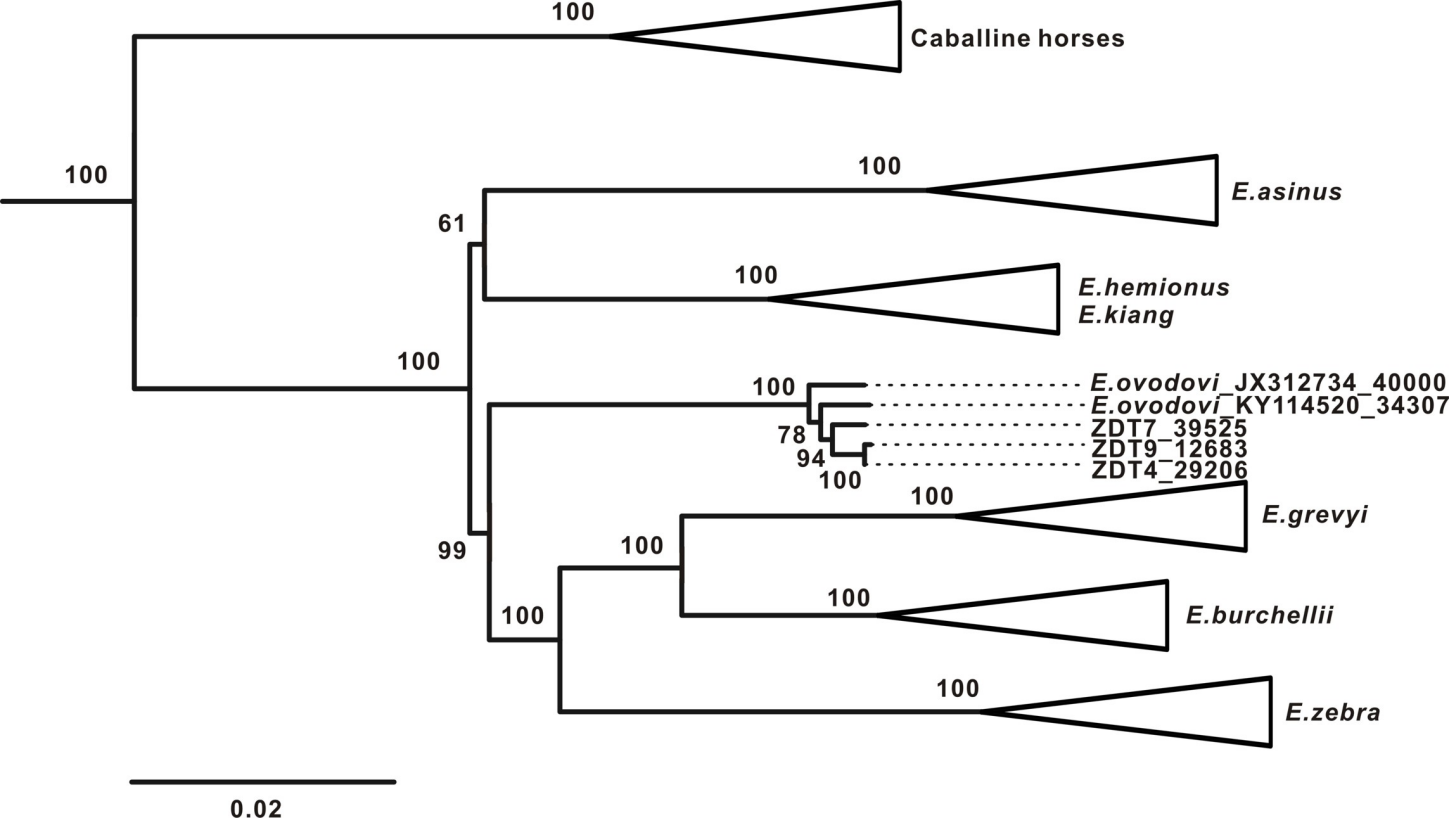
Maximum-likelihood phylogenetic tree of complete mitochondrial genomes of equids, rooted using the *Hippidion* clade as outgroup. Branch labels show the bootstrap values derived from 1000 replications. Ages of *E*. *ovodovi* specimens are indicated following the sample/accession number.

Maximum-likelihood phylogenetic analyses revealed two major clades: a caballine equid clade and a non-caballine equid clade (Fig 2). All the *E*. *ovodovi* individuals form a monophyletic group within the non-caballine equids clade, as suggested by previous analyses [3, 5–6]. Moreover, *E*. *ovodovi* forms the sister lineage to the zebras, which is in accordance with result obtained by Druzhkova *et al*. [6], with a high bootstrap support value of 99%. The non-caballine clade is also supported by a bootstrap support value of 100% and consists of the following groups: 1) all three zebra species, 2) all *E*. *ovodovi* samples, 3) *E*. *hemionus* and *E*. *kiang*, which cannot be distinguished using mitochondrial DNA, and 4) *E*. *asinus*. The monophyly of the hemionids (*E*. *kiang*, *E*. *asinus* and *E*. *hemionus*) lacks notable statistical support (61% bootstrap value).

**Fig 3:**
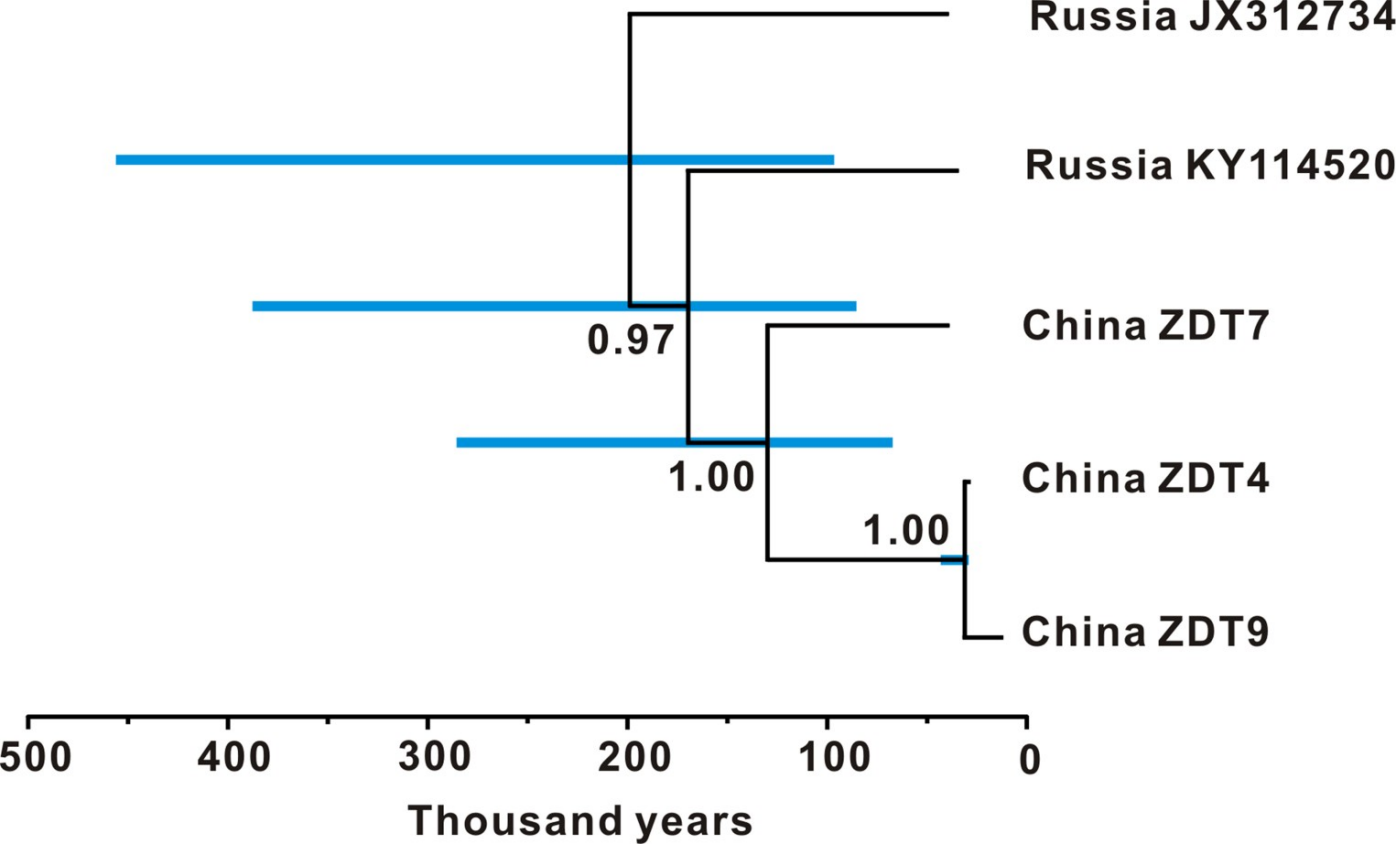
Maximum clade credibility tree of *E*. *ovodovi* haplotypes. Nodes heights are centred on the median posterior age estimate (x-axis) and tips on sample ages. Blue node bars show 95% credibility intervals of the coalescence times. Branch labels show posterior clade probabilities.

Bayesian phylogenetic analysis of *E*. *ovodovi* haplotypes in BEAST produced a well resolved phylogeny with high posterior confidence for all nodes (Fig 3). The Chinese haplotypes form a clade, while the two Russian haplotypes are not monophyletic, with sequence KY114520 sharing more recent ancestry with the Chinese clade than with the second Russian haplotype. The Chinese haplotypes coalesce within the last 130,000 years (95% credibility interval 67,000–286,000 years). The estimated coalescence of the Chinese clade and their Russian sister lineage is 170,000 years ago (95% credibility interval 86,000–388,000 years). All sampled haplotypes coalesce around 199,000 years ago (95% credibility interval 97,000–456,000 years).

**Fig 4:**
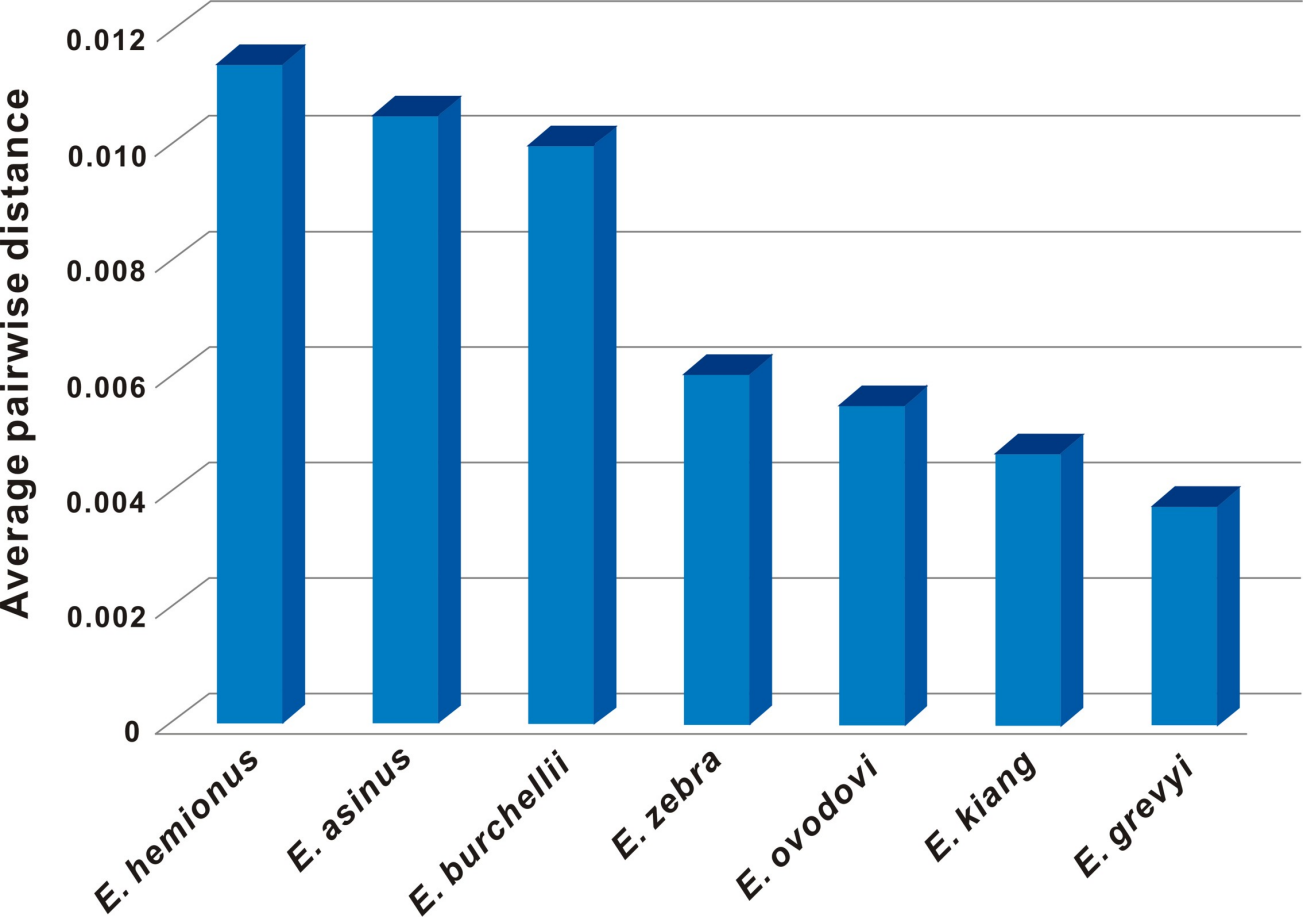
Average pairwise mitochondrial distances within non-caballine equid species.

Computing average pairwise distances showed that the numbers of nucleotide differences between Chinese *E*. *ovodovi* samples and Russia specimens are between 94 and 143, while the two Russia specimens differed from each other by 120 nucleotide sites, and there are only 5–72 nucleotide differences among the three Chinese samples (S4 Table). The averaged pairwise distance among *E*. *ovodovi* haplotypes (although based on only five sequences) is on an intermediate level compared to values for the other non-caballine *Equus* species (Fig 4).

## Discussion

### Morphological identification and geographic distribution of *E*. *ovodovi*

Our results provide clear evidence for the existence of *E*. *ovodovi* within the boundaries of present-day China during the Late Pleistocene, representing the first documented occurrence outside of present-day Russia.

In addition to *E*. *ovodovi*, three other species of the genus *Equus*, *E*. *hemionus*, *E*. *przewalskii*, and *E*. *dalianensis*, have been recorded from northern China, largely from Late Pleistocene strata from northeastern China [9, 11, 13, 15]. Among these species, *E*. *dalianensis* and *E*. *przewalskii* were assumed to be closely related and to share a recent common ancestor in *Equus beijingensis* [9]. Fossil remains suggested a wide distribution of *E*. *dalianensis* in northeastern China during the Late Pleistocene (Fig 1) [11–12, 14, 30–34], which would indicate both a temporal and geographic overlap with our *E*. *ovodovi* specimens (Fig 1).

Interestingly, the three *E*. *ovodovi* specimens analyzed in this study had been morphologically identified as *E*. *dalianensis*, suggesting that fragmentary remains of these species may be difficult to differentiate based on morphology. The holotype of *E*. *dalianensis* is an incomplete mandible with p2-m3. Other specimens include an upper jaw with P2-M3, metapodials and phalanxes. Its cheek teeth are similar to the extant *E*. *przewalskii* in morphology with deep ectoflexids penetrated into the isthmus and broad, U-shaped linguaflexids. *E*. *dalianensis* was a quite large sized horse. Its metacarpals are 231–249.5 mm long and 49–61 mm wide at the proximal extremity. Its metatarsals are 265–293 mm long and 50–60 mm wide at the proximal extremity. The holotype of *E*. *ovodovi* is a palatal fragment with P3-M3. Other specimens include lower cheek teeth and metapodials. Its deep ectoflexids and broad, U-shaped linguaflexids are similar to those of *E*. *dalianensis*. However, *E*. *ovodovi* is smaller in size and more gracile than *E*. *dalianensis*. For example, the metacarpal of *E*. *ovodovi* is about 230 mm long and 46 mm wide at the proximal extremity, and the metatarsals are 257.2–276.8 long and 41.5–48.3 mm wide at the proximal extremity. Thus, the metapodials of *E*. *ovodovi* are especially slender compared to those of *E*. *dalianensis*, and may thus provide useful skeletal elements for species identification. Nonetheless, incorrect prior assignment of the samples analysed in this study to *E*. *dalianensis* does highlight the utility of molecular approaches for unambiguous taxonomic assessments of ancient equid remains.

*E*. *ovodovi* was abundant in Khakassia, the Altai and alluvial deposits in the Altai piedmont lowlands in Late Pleistocene strata [4], which suggests that a key part of its distribution during the Late Pleistocene was in Siberia. There are no notable geographic barriers between southern Siberia and northeastern China. Thus, our study greatly extends the distribution area of *E*. *ovodovi* during the Late Pleistocene from Siberia to northeastern China. Furthermore, coalescence times and the lack of reciprocal monophyly of Russian and Chinese haplotype lineages is suggestive of gene flow or continuity of these populations during the Middle and/or Late Pleistocene. For a better understanding of the genetic relationships between the Siberian and the Chinese *E*. *ovodovi*, as well as between *E*. *ovodovi* and *E*. *dalianensis*, further genetic studies including more specimens and nuclear DNA markers, will be required.

### Phylogenetic position and genetic diversity of *E*. *ovodovi*

Our phylogenetic analysis strongly supports the position of *E*. *ovodovi* within the non-caballine equid clade (Fig 2), as found by several previous studies [3, 5–6]. However, previous studies could not resolve relationships within the non-caballine equids clade. Orlando *et al*. [3] and Vilstrup *et al*. [5] suggested a basal position of *E*. *ovodovi* in the non-caballine equids clade, while Druzhkova *et al*. [6] suggested that *E*. *ovodovi* is more closely related to the zebras than to *E*. *asinus*, *E*. *hemionus* and *E*. *kiang*. Our phylogenetic analyses support the relationship identified by the latter study with strong bootstrap support.

Although based on only five specimens, calculation of pairwise distances among haplotypes revealed similar levels of diversity as several other *Equus* species, and even slightly higher than in *E*. *kiang* and *E*. *grevyi*. Thus, this preliminary analysis suggests that Late Pleistocene populations of *E*. *ovodovi*, representing the last surviving representatives of the subgenus *Sussemionus*, did not show especially reduced genetic diversity in the period prior to their final extinction. Increased sampling of individuals and genetic loci would be desirable to more robustly test this hypothesis.

### The latest occurrence of *E*. *ovodovi*

Our specimens were excavated from northeastern China at almost the same latitude as the *E*. *ovodovi* localities known so far from Russia. By comparing the species of Late Pleistocene fauna that historically existed in northeastern China and south Siberia, researchers found that, in general, mammalian faunal assemblages of these two regions were highly similar [35]. This suggests that members of a species from the two respective regions may have formed a single large population, or existed as structured subpopulations connected by high levels of gene flow. Northeastern China is the highest latitudinal region in China, which was deeply affected by the strong cold stream from Siberia. Pollen analyses and mammal fauna species reflected a dry and cold climate during the Late Pleistocene [35–36]. A large number of animal species adapted to the cold and dry climate have been found in Late Pleistocene deposits in this region [12, 37–39]. *E*. *ovodovi* was therefore likely adapted to an environment with moderately dry climatic conditions and steppe landscapes [4, 40].

One of our specimens was dated at 12,770–12,596 cal. yBP, which is substantially younger than the previous latest date of 32,000 yBP [6], and represents the latest occurrence of undoubted material of this species so far reported. Therefore, our study extends the time range of *E*. *ovodovi* to the Pleistocene-Holocene boundary. The terminal Pleistocene date for the existence of *E*. *ovodovi* and the climate record [40–41] suggest that this fossil species vanished from its Pleistocene habitats when the climate became more humid and warmer than during previous time periods.

## Conclusion

In summary, our study verifies the existence of *E*. *ovodovi* in northeastern China during the Late Pleistocene until at least the turning point of the Pleistocene to Holocene. However, the details of the evolutionary history of *E*. *ovodovi* still remain unresolved, such as its full geographical and temporal distribution, evolutionary origin, and the process of its extinction. Therefore, more biomolecular and morphological analyses are needed for a better understanding of this recently discovered ancient horse species.

## Supporting information

S1 Dataset Manuscript data. (DOCX)

S1 File The BEAST input xml file is included as supplementary information. (XML)

S1 Table Long range PCR primer pairs used in this study. (DOCX)

S2 Table Sequences used in this study to investigate the genetic diversity of the non-caballine equid members. (DOCX)

S3 Table Sequencing statistic results for NGS reads. (DOCX)

S4 Table Number of nucleotide differences between *E*. *ovodovi* samples based on complete mitochondrial genomes. (DOCX)
