# Supplementary material for: Molecular identification of late and terminal Pleistocene Equus ovodovi from northeastern China
Source: PLoS One. 2019 May 16;14(5):e0216883. doi: 10.1371/journal.pone.0216883 (PMC6522033; doi:10.1371/journal.pone.0216883)
Supplement: S1 Dataset — (DOCX) [file pone.0216883.s001.docx]

Sample no. ZDT7

GTTAATGTAGCTTAATAATATAAAGCAAGGCACTGAAAATGCCTAGATGAGTATTCCCACTCCATAAACACATAGGCTTGGTCCTAGCCTTTTTATTGGTTATTAATAGAATTACACATGCAAGTATCCGCGCCCCAGTGAGAATGCCCTCTAAATCGCACTCCACGATCAAAAGGAGCAGGTATCAAGCACACTAGAAAGTAGCTTATAACACCTTGCTCAGCCACACCCCCACGGGACACAGCAGTGATAAAAATTAAGCCATGAACGAAAGTTCGACTAAGTTATATTAAATTAGGGTTGGTAAAATTCGTGCCAGCCACCGCGGTCATACGATTAACCCAAATTAATAAAATCCCGGCGTAAAGCGTGTCAAAGACCTTACACCAAAATAAAGTTAAAACCCAGTTAAGCTGTAAAAAGCTACAACCAAAGTAAAATAGACTACGAAAGTGACTTTAATACCTCTGACCACACGATAGCTAAGACCCAAACTGGGATTAGATACCCCACTATGCTTAGCCCTAAACTAAAATAGCTCATCACAACAAAGTTATTCGCCAGAGTACTACTAGCAACAGCCTAAAACTCAAAGGACTTGGCGGTGCTTTACATCCCTCTAGAGGAGCCTGTTCCATAATCGATAAACCCCGATAAACCCCACCATCCCTTGCTAATTCAGCCTATATACCGCCATCTTCAGCAAACCCTAAACAAGGCACCAAAGTAAGCACAATCATCCAACATAAAAACGTTAGGTCAAGGTGTAGCCCATGGGATGGAGAGAAATGGGCTACATTTTCTACTCTAAGAACAAAAACTTAACCCAAACGAAAGTCTCTATGAAATTGGAGACCGAAGGAGGATTTAGCAGTAAATTAAGAATAGAGAGCTTAATTGAATCAGGCCATGAAGCGCGCACACACCGCCCGTCACCCTCCTTAAATATCACAAACCACAATTTTAACACAAAACCGTGGCCCAAACATATGAAAGGAGACAAGTCGTAACAAGGTAAGTATACCGGAAGGTGTACTTGGATAACCAAAGTGTAGCTTAAACAAAGCATCCAGCTTACACCTAGAAGATTTCACTCAGAATGAACACTTTGAACTAAAGCTAGCCCAAACGACATCCAACTCAACTACCCTTAGTCACTTAACTAAAACATTCACTAAACCATTAAAGTATAGGAGATAGAAATTTTAACTTGGCGCTATAGAGAAAGTACCGTAAGGGAACGATGAAAGATACATTAAAAGTACCAAACAGCAAAGCTTACCCCTTTTACCTTTTGCATAATGACTTAACTAGAATAAACTTAGCAAAGAGAACTTAAGCTAAGCACCCCGAAACCAGACGAGCTACCTACGAACAGTTACAAAGAACCAACTCATCTATGTCGCAAAATAGTGAGAAGATTCATAGGTAGAGGTGAAAAGCCCAACGAGCCTGGTGATAGCTGGTTGTCCAGAAACAGAATCTCAGTTCAAATTTAAGTTTACCTAAAAACCACCCAATTCTAATGTAAACTTAAATTATAATCTAAAAAGGTACAGCTTTTTAGATACAGGATACAACCTTTATTAGAGAGTAAGAATAAGATAATCCCATAGTTGGCTTAAAAGCAGCCATCAATTAAGAAAGCGTTCAAGCTCAACATCACATTTATCTTAATCCCAAAAATAAACTCAAACTAACTCCTAATCTTATACTGGACTATTC-TATCAACACATAGAAGCAATAATGTTAATATGAGTAACAAGAATTATTTCTCCTTGCATAAGCCTATATCAGAACGAATACTCACTGATAGTTAACAACAAAATAGACACAACCCAAAAACTAACCACCTATTTAAATTATTGTTAACCCAACACAGGCATGCGCCCATAAGGAAAGATTAAAAGAAGTAAAAGGAACTCGGCAAACACAAACCCCGCCTGTTTACCAAAAACATCACCTCTAGCATTTCCAGTATTAGAGGCACTGCCTGCCCAGTGACATCTGTTTAAACGGCCGCGGTATCCTAACCGTGCAAAGGTAGCATAATCACTTGTTCTCTAAATAGGGACTTGTATGAATGGCCACACGAGGGTTTTACTGTCTCTTACTTCCAATCAGTGAAATTGACCTTCTCGTGAAGAGGCGAGAATAACCAAATAAGACGAGAAGACCCTATGGAGCTTTAATTAACTGATTCACAAAAAACAACATACAAACCTAACCCTCAGGGACAACAAAACTTTTGATTGAATCAGCAATTTCGGTTGGGGTGACCTCGGAGAACAAAACAACCTCCGAGTGATTTAAACCTAGACTAACCAGTCAAAATACATAATCACTTATTGATCCAAACCATTGATCAACGGAACAAGTTACCCTAGGGATAACAGCGCAATCCTATTCCAGAGTCCATATCGACAATTAGGGTTTACGACCTCGATGTTGGATCAAGACATCCTAATGGTGCAACCGCTATTAAGGGTTCGTTTGTTCAACGATTAAAGTCTTACGTGATCTGAGTTCAGACCGGAGTAATCCAGGTCGGTTTCTATCTATTCTACACTTTTCCCAGTACGAAAGGACAAGAAAAGTAGGGCCCACTTTACAAGAAGCGCCCTTAAACTAATAGATGACATAATCTAAATCTAACTAATTTATAAACCTCACCGCCCTAGAACAGGGCTCGTTAGGGTGGCAGAGCCCGGAAATTGCATAAAACTTAAACCTTTATACCCAGAGGTTCAATTCCTCTCCCTAACAACATGTTCATAATTAACGTTCTCCTCCTAATTGTCCCAATCCTGCTCGCCGTAGCATTCCTCACACTAGTTGAACGAAAAGTCTTAGGCTACATACAACTTCGCAAAGGACCCAACATCGTAGGCCCCTATGGCCTACTACAACCCATTGCCGATGCCCTCAAACTATTTATCAAAGAACCACTACAACCACTAACATCATCGACATCCATATTTATCATCGCACCAATCCTAGCCCTCACCCTAGCCTTAACTATATGAATCCCTCTCCCCATACCGTACCCACTAATTAACATAAACCTAGGAATTCTATTCATATTAGCCATATCCAGCCTAGCTGTCTACTCAATCCTTTGATCAGGATGAGCCTCAAACTCAAAATACGCTCTAATTGGAGCCCTACGAGCAGTAGCACAAACCATCTCATATGAAGTAACTCTAGCAATCATTCTACTCTCAGTCCTCCTAATAAGCGGATCATTCACACTATCAACACTTATCACTACCCAAGAATACTTATGATTAATCTTCCCATCATGACCCTTAGCCATAATATGATTTATCTCAACATTAGCCGAAACCAACCGAGCTCCATTTGACCTAACAGAAGGAGAATCAGAACTCGTCTCCGGATTCAACGTTGAATACGCAGCCGGCCCATTCGCTCTATTCTTCCTAGCAGAGTATGCAAACATCATCATAATGAACATCTTCACAACAACTCTATTTCTAGGAGCATTTCACAACCCCTACCTACCAGAACTCTACTCAATTAATTTTACCATCAAAGCCCTCCTTCTAACATGTTCCTTCCTATGAATCCGAGCATCCTACCCGCGATTCCGATATGACCAACTTATACACCTCCTATGAAAAAACTTTCTACCACTCACACTAGCCCTCTGCATATGACATGTCTCGCTACCAATCATACTATCCAGCATCCCACCACAAACATAAGAAATATGTCTGACAAAAGAGTTACTTTGATAGAGTAAAACATAGAGGTTCAAACCCTCTTATTTCTAGAACCACAGGAATTGAACCTGCTCCTGAGAATTCAAAATCCTCCGTGCTACCAAATTACACCATGCCCTACAAGTAAGGTCAGCTAAATAAGCTATCGGGCCCATACCCCGAAAATGTTGGATTACACCCTTCCCGTACTAATAAACCCCCTTATCCTCACAATTATCCTAATAACAGTTTTTCTAGGAACTATAATCGTCATAATAAGCTCACACTGACTAATAATCTGAATCGGATTCGAAATAAATCTACTAGCCATTATCCCCATCCTAATAAAAAAATACAGCCCCCGAGCCATAGAAGCCTCCACCAAATACTTCCTAACCCAAGCCACCGCATCTATACTCCTCATAATAGCAATTATCATCAACCTGATGCACTCAGGCCAATGAACAATCACAAAAATTTTCAACCCCACAGCATCCATCATCATAACTTCAGCCCTTGCCATAAAACTTGGACTCACACCATTCCACTTCTGAGTGCCCGAAGTCACACAGGGCATCTCACTAACATCAGGCCTCATCCTACTCACATGACAAAAACTAGCCCCAATATCAATCCTATATCAAATCTCACCCTCAATCAACCTGAACATCTTACTTACCATAGCCGTACTGTCAATCCTGGTAGGAGGCTGAGGTGGTCTCAACCAAACCCAACTACGAAAAATTATAGCATACTCGTCGATCGCACATATAGGATGAATAATGGCTGTCCTGGCATACAACCCAACACTGACAATACTAAACATACTAATTTACATTGTAATAACACTCACAATATTCATACTGTTTATCCACAGCTCCTCCACTACAACACTATCACTCTCACACACATGAAACAAAACACCTCTAACCACCACACTAATTCTAATCACCTTGCTATCTATAGGAGGCCTCCCCCCGCTATCAGGATTCATACCCAAATGAATAATCATCCAAGAACTCACCAAAAACAGCAGCATCATTCTCCCCACTCTAATGGCTATCATAGCACTACTCAATCTCTACTTCTACATACGACTAACCTATTCCACCTCCCTAACCATATTCCCATCCACAAACAACATAAAAATAAAATGACAATTCGAAGCCAAACAAATTACCCTCCTACCCCCATTAATCGTCGCATCTTCCCTACTCCTCCCCCTAACCCCCATACTATCAATTCTGGACTAGGAATTTAGGTTAGCATCCAGACCAAGAGCCTTCAAAGCTCTAAGCAAGTATATTCACTTAATTCCTGCACACTAAGGACTGCAAGACTCTATCTCACATCAATTGAACGCAAATCAAACACTTTCATTAAGCTAAGCCCTTACTAGATTGGTGGGCTATCATCCCACGAAACTTTAGTTAACAGCTAAACACCCTAATCAACTGGCTTCAATCTACTTCTCCCGCCGCCTAAAAAAAAAGGCGGGAGAAGCCCCGGCAGAATTAAAGCTGCTCCTTCGAATTTGCAATTCAATGTGAAATTCACCACGGGACTTGATAAGAAGAGGATTCCAACCCCTGTCTTTAGATTTACAGTCTAATGCTTACTCAGCCATCTTACCTATGTTCATCAACCGCTGACTATTTTCGACTAACCACAAAGACATCGGCACTCTGTACCTCCTATTCGGCGCTTGAGCTGGAATAGTAGGAACCGCCCTAAGCCTCCTAATCCGTGCTGAATTAGGCCAACCTGGGACCCTACTAGGAGATGACCAGATCTACAATGTTATTGTAACCGCCCACGCATTCGTAATAATTTTCTTTATAGTCATACCCATTATGATCGGAGGATTTGGGAACTGATTAGTCCCCTTAATAATTGGAGCACCTGACATAGCTTTCCCCCGAATAAACAACATAAGCTTCTGATTACTTCCCCCATCATTCCTGCTCCTTCTTGCTTCCTCAATAATTGAAGCAGGCGCTGGAACAGGCTGAACCGTATACCCTCCCCTAGCTGGGAATCTAGCGCATGCAGGGGCCTCTGTTGACTTAACCATTTTCTCTCTCCACCTAGCTGGTGTATCTTCAATCCTAGGTGCCATCAATTTCATTACTACAATCATCAACATAAAACCGCCAGCCCTATCCCAATATCAAACTCCTCTATTCGTTTGATCCGTCCTTATTACGGCAGTACTCCTTCTTCTAGCCCTTCCAGTCCTAGCAGCAGGCATTACCATGCTTCTCACAGACCGTAACCTAAACACCACCTTCTTCGACCCTGCAGGAGGAGGAGACCCAATCCTTTACCAACACCTATTCTGATTCTTCGGACACCCCGAAGTCTACATTCTCATCCTGCCAGGTTTTGGTATAATCTCACACATCGTTACATATTATTCAGGTAAAAAGGAACCTTTTGGCTACATGGGTATAGTGTGAGCTATAATATCCATTGGCTTTCTAGGCTTCATCGTATGAGCTCACCACATGTTTACAGTAGGTATAGACGTCGATACACGAGCATACTTCACATCAGCTACCATAATCATCGCCATCCCTACTGGTGTAAAAGTATTCAGCTGACTAGCCACCCTACACGGAGGAAATATCAAATGATCCCCAGCTATACTCTGAGCTCTAGGCTTCATCTTCTTATTCACAGTAGGAGGCCTAACAGGCATCGTCTTGGCTAATTCATCCCTAGATATTGTTCTCCATGATACTTATTATGTAGTAGCACATTTCCACTATGTCCTGTCCATAGGAGCAGTCTTCGCCATTATAGGAGGATTTGTTCACTGATTCCCTCTATTCTCAGGATATACACTCAACCAAACCTGAGCAAAAATCCACTTTACAATTATATTCGTAGGGGTCAATATAACTTTCTTCCCACAACACTTCCTTGGCCTCTCAGGAATACCACGACGCTACTCTGATTACCCAGACGCATACACAACATGAAACACCATCTCATCCATAGGATCTTTCATCTCACTCACAGCAGTAATACTAATAATCTTCATGATTTGGGAAGCATTCGCATCTAAACGAGAAGTGTCTACAGTAGAATTAACCTCAACTAACCTAGAATGACTACACGGATGCCCCCCACCATACCACACATTCGAAGAACCCGCTTATGTAAACCTAAAATAAGAAAGGAAGGAATCGAACCCCCTCTAACTGGTTTCAAGCCAATATCATAACCACTATGTCTTTCTCCATTAAACGAGGTATTAGTAAAAATTACATAACTTTGTCAAAGTTAAATTATAGGTTGAACCCCTATATGCCTCTATGGCCTACCCCTTCCAATTAGGATTCCAAGACGCAACATCCCCCATTATAGAAGAACTCCTACACTTCCATGACCACACGCTAATAATCGTATTCCTAATTAGCTCTCTAGTATTATACATTATCTCATCAATACTAACGACTAAATTAACCCACACTAGCACCATAGACGCCCAAGAAGTAGAAACAATTTGAACAATTCTACCAGCCATTATCCTTATTCTAATTGCCCTCCCATCCCTACGAATTCTATATATAATAGATGAAATCAACAACCCATCTCTTACGGTCAAAACAATAGGCCACCAATGATACTGAAGTTACGAGTATACCGATTACGAGGACCTGACCTTCGATTCCTACATGATCCCCACATCAGACCTAAAACCAGGAGAATTACGTCTTCTAGAAGTCGACAACCGAGTGGTTCTCCCTATGGAAATAACTATCCGAATACTAATCTCATCCGAAGACGTCCTACACTCATGAGCTGTCCCCTCCCTAGGCCTAAAAACAGACGCCATCCCTGGGCGCCTAAATCAAACAACTCTCGTAGCCTCCCGACCAGGTCTTTACTACGGCCAATGTTCGGAGATCTGCGGATCAAACCACAGCTTCATACCAATTGTCCTTGAACTAGTCCCACTGAAACACTTCGAAGAATGATCTGCATCAATATTATAAAATCACTAAGAAGCTATTATAGCGTTAACCTTTTAAGTTAGAGACTGAGGGTTCAACCCCCTCCCTAGTGATATGCCACAGTTGGATACATCAACATGATTTATTAATATCGTCTCAATGATCTTAACTCTATTTATTGTATTTCAACTAAAAATCTCAAAGCACTCTTACCCAACACACCCAGAAGCAAAAACAACTAAAATAACCAAACACTTCACCCCTTGAGAATCAAAATGAACGAAAATCTATTCGCCTCTTTCGCTACCCCAACAGTAATAGGCCTCCCTATTGTGATTCTAATCATCATATTCCCCAGCATCCTATTCCCTTCATCCAACCGACTAATCAACAACCGCCTAATCTCAATTCAACAATGACTAGTCCAACTTACATCAAAACAAATAATAGCTATTCACAACAACAAAGGACAAACCTGAACCCTCATACTCATGTCACTAATCCTATTCATTGGCTCGACAAACTTATTGGGCCTACTACCCCATTCATTTACACCAACAACACAACTATCAATAAATCTAGGCATAGCCATCCCCCTGTGAGCAGGAACAGTATTCATAGGCTTCCGTCATAAAACAAAAGCAGCCCTAGCTCACTTTCTGCCTCAAGGAACACCCATTTTCCTCATTCCCATGCTAGTAATTATTGAGACTATCAGCCTATTTATTCAACCTGTAGCTCTAGCCGTACGGCTAACTGCTAACATTACTGCCGGACATCTTCTAATTCACCTTATCGGAGGAGCAACACTAGCCCTCATAAATATTAGCCCCTCAACAGCCCTTATTACATTTATTATCCTAATTCTGCTGACTATCCTCGAATTTGCAGTAGCCATAATCCAAGCCTACGTATTCACTCTTCTAGTAAGCCTCTACCTACATGATAACACCTAATGACCCACCAAACCCACGCCTACCACATAGTAAATCCCAGCCCATGACCACTTACAGGAGCCCTATCAGCCCTCCTAATAACATCAGGATTAGCCATGTGATTCCACTTTAACTCAACTCTACTTCTAGCTTTAGGACTATTAACCAACATCCTCACCATATATCAATGATGACGAGACATCATCCGAGAAAGCACATTTCAAGGCCACCACACATTAATCGTCCAAAAAGGACTCCGATACGGCATAATCCTTTTCATCATCTCAGAAGTCTTCTTCTTCTCTGGCTTCTTTTGAGCCTTCTACCACTCAAGCCTAGCTCCCACACCCGAACTAGGCGGCTGCTGACCACCCACAGGTATCCACCCCTTAAACCCCCTAGAAGTCCCCTTACTCAACACCTCAGTGCTCCTAGCATCTGGGGTCTCTATCACCTGAGCCCACCATAGCCTGATAGAAGGGAACCGTAAAAACATGCTCCAGGGCCTATTCATCACAATCTCACTAGGCGTATACTTCACTCTCCTCCAAGCCTCAGAATACTACGAAGCCTCATTTACCATTTCAGATGGGGTGTATGGATCGACATTTTTCGTGGCAACAGGATTCCACGGACTACACGTAATCATCGGATCCACCTTCCTTATCGTATGCTTCCTACGCCAACTAAAATTCCACTTTACATCCAGCCACCATTTCGGATTCGAAGCAGCCGCTTGATACTGACACTTCGTCGACGTAGTTTGACTATTCTTATATGTCTCCATCTATTGATGAGGATCCTATTCTTTTAGTATTGACCAGTACAATTGACTTCCAATCAATCAGCTTCGGTACAACCCGAAAAAGAATAATAAACCTCATACTAACACTCCTCACCAACACATTACTAGCCTCACTACTCGTACTCATCGCATTCTGACTACCGCAACTAAATATCTATGCAGAAAAAACCAGCCCATACGAATGCGGATTTGACCCAATAGGGTCAGCACGCCTCCCTTTCTCAATAAAATTTTTCTTAGTGGCCATTACATTTCTGCTGTTCGACCTAGAAATCGCCCTCCTGCTACCCCTTCCATGAGCATCCCAGACAACTAACCTGAACACCATACTTATCATAGCACTAATCCTAATCTCTCTCCTAGCTATCAGCCTGGCCTATGAATGAACCCAAAAAGGACTAGAATGAACTGAGTATGGTAATTAGTTTAAACTAAAACAAATGATTTCGGCTCATTAAACTATGATTAACTTCATAATTACCAACATGTCACTAGTCCACATTAATATCTTCCTAGCATTCACAGTCTCCCTCGTAGGCCTACTAATGTACCGATCCCACCTAATATCCTCACTCCTATGCCTAGAAGGGATAATACTGTCACTATTCGTCATAGCAACCATAATAGTCCTAAACACCCATTTCACACTAGCCAGCATAATACCCATTATCCTACTAGTATTCGCTGCCTGCGAAGCAGCATTAGGGCTATCCTTACTAGTCATAGTCTCCAATACTTATGGAGTGGACCACGTACAAAACCTTAACCTCCTCCAATGCTAAAAATCATCATTCCCACAATCATACTAATACCCCTCACATGACTATCAAAAAAGAACATAATCTGAATTAACACCACAACCTACAGTCTATTAATCAGCCTCATCAGCCTATCCCTCCTAAATCAGCCTAGCAACAATAGCCTAAACTTCTCACTAATATTCTTCTCTGACCCCCTATCAGCTCCACTACTAGTACTAACAACATGACTACTACCACTAATACTCATAGCCAGCCAACATCACCTATCCAAAGAACCACTAATCCGAAAAAAACTCTACATCACCATGCTAACCATACTTCAAACTTTCCTAATTATAACTTTTACCGCCACAGAACTAATCTCCTTCTACATCTTATTCGAAGCCACATTAGTTCCAACACTAATTATCATCACCCGCTGAGGCAACCAAACAGAACGCCTAAACGCAGGCCTCTACTTTCTATTCTACACACTAATAGGCTCTCTCCCACTTTTAGTCGCACTAATCTCCATCCAAAACCTAACAGGCTCACTAAACTTCCTACTAATCCAATACTGAAACCAAACACTACCCGACTCCTGATCTAATATCTTCCTATGATTAGCATGTATAATAGCATTTATAGTTAAAATGCCCCTATATGGTCTCCACCTCTGACTTCCAAAAGCCCATGTAGAAGCCCCAATCGCTGGATCCATAGTACTAGCAGCCATCCTACTAAAACTAGGAGGCTACGGAATACTGCGAATTACAACAATATTAAACCCTCAAACCAACTTTATAGCCTACCCTTTCCTTATACTATCTCTATGAGGAATAATCATAACTAGCTCCATCTGTTTACGACAAACTGATCTAAAATCACTTATTGCATACTCCTCTGTCAGTCATATAGCCCTAGTAATTGTAGCCGTCCTTATCCAAACACCATGAAGCTATATAGGGGCCACAGCCCTAATAATCGCCCACGGTCTTACGTCATCAATACTATTCTGCCTAGCAAACTCAAACTACGAACGCACTCACAGCCGAACCATAATCCTAGCCCGCGGACTTCAAACACTCCTCCCCCTCATAGCAGCCTGATGACTATTAGCCAGCTTAACCAACCTGGCCCTCCCTCCCAGCATTAACCTAATCGGAGAACTATTCGTAGTAATATCGTCATTCTCATGATCAAATATTACTATCATCCTCATGGGAACTAACATCACCATCACCGCTCTCTACTCCCTCTATATACTAATCACCACACAACGAGGAAAATACACACACCACATCAATAACATCAAACCCTCATTTACACGAGAAAACGCACTCATAGCCCTCCACATAACTCCCCTACTACTCCTATCACTTAACCCTAAAATCATCCTAGGCTTTACGTACTGTAAATATAGTTTAACAAAAACACTAGATTGTGGATCTAGAAATAGAAACTCGACATTTCTTATTTACCGAGAGAGTATGCAAGAACTGCTAATTCATGCCTCCATGTCTGACAAACATGGCTCTCTCCCAAACTTTTAAAGGATAGGAGCTATCCGTTGGTCTTAGGAACCAAAAAATTGGTGCAACTCCAAATAAAAGTAATCAACATATTCTCCTCCCTCATACTAGTTTCACTATCAGTATTAACCCTGCCTATCATATCATCAATCCTCAATACCCACAAAAACAACACGTACCCACATCACGTAAAAAATATTATCTCATATGCCTTCATTACCAGCCTAATTCCCACCATAATATTCATCCACTCTGGACAAGAAACAATCATCTCAAACTGACACTGAGTAACTATACAAACCCTCAAACTATCTCTAAGCTTCAAACTAGACTACTTCTCAATAGTCTTCGTACCGGTAGCCCTATTCGTAACATGATCTATCATAGAATTCTCTTTATGATACATGCACTCAGATCCTTACATCACCCGATTTTTTAAATACCTACTCACATTCCTCATTACCATAATAATCCTAGTCACAGCCAATAACCTCTTCCAACTGTTCATTGGATGAGAAGGAGTAGGCATCATATCATTCTTACTAATCGGATGATGGTATGGTCGAACAGACGCCAACACTGCGGCCCTCCAAGCAATCCTATATAACCGCATCGGAGATATTGGCTTCATCATAGCCATGGCCTGATTCCTATTCAACACCAACACATGAGACCTACAACAAATCTTCATACTTGACCCCAACCTCACCAACCTCCCACTCCTAGGCCTTCTCCTAGCCGCAACTGGTAAATCTGCCCAATTCGGACTCCACCCATGACTTCCCTCAGCCATAGAAGGCCCCACACCAGTCTCAGCCCTACTCCACTCCAGCACAATAGTTGTAGCAGGCGTCTTCCTACTAATCCGTTTCCACCCACTAATAGAAAACAACAAAACCATCCAGTCACTCACCCTATGCCTAGGAGCTATCACCACACTATTCACAGCAATCTGTGCACTCACCCAAAACGACATCAAAAAAATCATCGCCTTCTCCACCTCCAGCCAACTAGGTCTGATAATCGTAACCATCGGCATTAATCAGCCCTACCTAGCATTCCTTCACATCTGCACTCACGCATTCTTCAAAGCCATACTATTCATATGCTCCGGATCCATTATCCACAGCCTAAATGACGAGCAGGACATCCGAAAAATAGGCGGGCTGTTTAATGCAATACCTTTCACCACCACATCCCTAATTATTGGCAGCCTCGCACTCACCGGCATACCTTTCCTCACAGGCTTTTACTCCAAAGACCTCATCATCGAAACCGCCAACACATCGTACACCAACGCCTGAGCCCTACTAATAACACTCATCGCCACATCCCTCACAGCTGTCTACAGCACCCGAATCATCTTCTTCGCACTCCTAGGACAACCTCGCTTCCTCCCCTTAACTTCAATCAACGAAAATAATCCTTTCCTAATTAACTCCATTAAACGCCTCTTAATTGGCAGCATTTTCGCCGGATTCTTTATCTCTAACAATATCTACCCCACAACCGTCCCAGAAATAACCATACCTACCTACATAAAACTTACCGCCCTCGCAGTAACCATCCTAGGGTTTACACTAGCCCTAGAACTAAGCCTAATTACGCATAATCTAAAACTAGGACACCCCACCAACATATTTAAATTCTCCAACCTCCTAGGTTACTACCCAACAATCATACATCGACTCCCACCGCTCGCAAACCTATCAATAAGCCAAAAATCAGCATCACTTCTACTAGACTCAATCTGACTAGAAAGCATCCTACCAAAATCCATCTCCCAATTCCAGATAAAAACCTCAATCCTAATTTCCACCCAAAAAGGCCAAATCAAGCTATATTTCCTCTCATTCCTTATCACCCTCACCCTAAGCATACTACTTTTTAATCTCCACGAGTAACCTCTAAAATTACCAAGACTCCAACAAGCAACGACCAACCAGTCACAATCACAACCCAAGCCCCATAACTATACAATGCAGCAACCCCCATAATCTCCTCACTAAACACCCCAGAATCTCCAGTATCATAGATAGCTCAATCCCCTACACCACTAAACTTAAACACTACCCCCACTTCCTCACTCTTCAGAACATATAAAACCAGCATAACCTCTATCAACAACCCCAAAAGAAACACCCCCATAACAGTCGTATTAGACACCCACACCTCAGGGTATTGCTCAGTAGCCATAGCCGTTGTATAACCAAAAACAACCAACATTCCCCCCAAATAAATCAAAAACACCATCAACCCTAAAAAGGACCCTCCAAAATTCATAATGATGCCACAACCAACCCCTCCACTTACAATCAACACTAAACCCCCATAAATAGGCGAAGGTTTTGAAGAAAAACCTACAAAACCAATAACAAAGATAACGCTCAAAATAAATACAATATATGTCATCATTATTCCTACGTGGAATCTAACCACGACTAATGACATGAAAAATCATCGTTGTATTTCAACTATAAGAACACTAATGACAAATATCCGAAAATCTCACCCGCTAATTAAAATCATCAATCACTCCTTCATCGACCTACCAGCCCCCTCAAACATTTCATCATGATGAAACTTTGGCTCCCTCCTAGGAATCTGCCTAATCCTCCAAATCCTTACAGGTCTATTCCTAGCCATACACTACACATCAGACACAACAACCGCCTTCTCATCCGTTACTCACATCTGCCGAGATGTCAACTACGGATGAATCATTCGCTACCTCCACGCTAACGGAGCATCCATATTTTTCATCTGCCTCTTCATCCACGTAGGACGCGGCCTCTACTATGGCTCCTACACATTCCTAGAAACATGAAACATTGGAATTATCCTACTTTTCACAGTAATAGCCACAGCATTCATAGGCTATGTCCTACCATGAGGCCAAATATCCTTCTGAGGAGCAACAGTCATCACAAACCTCCTATCAGCAATCCCCTACATCGGCACTACCCTCGTCGAATGAATCTGAGGTGGATTCTCAGTAGACAAAGCCACCCTTACCCGATTTTTTGCCTTCCACTTTATTCTACCCTTTATCATCACAGCCCTGGTAGTCGTCCATCTACTATTCCTCCACGAAACAGGATCCAACAACCCCTCAGGAATCCCATCTGACATAGACAAAATCCCATTCCACCCATACTACACAATTAAAGACATCCTAGGACTCCTCCTCCTAATCCTACTCCTACTGACCCTAGTACTATTCTCCCCTGACCTCCTAGGAGACCCGGACAACTACACCCCAGCTAACCCTCTTAGCACTCCCCCTCATATTAAGCCAGAATGATACTTCCTATTTGCCTACGCCATCCTACGCTCTATTCCCAACAAACTAGGCGGCGTATTAGCCCTTATCCTTTCCATCTTAATCTTAGCACTCATCCCCACTCTACACATATCAAAACAACGAAGCATAATATTCCGACCTCTTAGTCAATGCGTGTTCTGACTCTTAGTAGCAGACTTACTGACACTAACATGAATCGGCGGCCAGCCAGTGGAACACCCATACGTAATTATCGGCCAACTGGCCTCAATCCTCTACTTCTCCCTAATTCTCATCTTCATACCACTCGCAAGCACCATCGAAAACAATCTTCTAAAGTGAAGAGTCCCTGTAGTATATCACATATTACTCTGGTCTTGTAAACCAGAAAAGGGGGAAACATTCCCCCCAAGGACTGTCAAGGAAGAAGCCCCAGCTCCACCATCAGCACCCAAAGCTGAAATTCTACTTAAACTATTCCTTGAATTTCCTCCCCTAAACGACAACAATTCATCCTCGTGTGCTATGTCAGTATTAAAACATACCCTACGCAATATCGTACATAACCCAACACACAATATCCTATTAACGCCCTATGTACGTCGTGCATTAAATTGTTTACCCCATGAATAATAAGCATGTACATAATATCATTTACTTTACATGAGTACATTATATTATTAATCGTGCATACCCCATTCAAGTCAAATCATTTCCAGTCAACACGCATATCACCGCCCATATTCCACGAGCTCAATCACCAAGCCGCGGGAAATCAGCAACCCTTCCAATCATGTGTCCCAATCCTCGCTCCGGGCCCATTCGAGTGTGGGGGTTTCTACAGTGAAACTATACCTGGCATCTGGTTCTTTCTTCAGGGCCATCTCACCCAACCTCGCCCATTCTTTCCCCTTAAATAAGACATCTCGATGGACTAATGACTAATCAGCCCATGCTCACACATAACTGTGGTTTCATGCATTTGGTATCTTTTTTATTTTGGGGATGCTATGACTCAGCTATGGCCGTCAGAGGCCCCGACGCAGTCAATTAAATTGAAGCTGGACTTAAATTGAACGTTATTACTCCGCATACAACAACCATAAGGTGTTATTCAGTCCATGGTAACAGGACATAGAGAATAAGCAGCACNNNNCCACACCAAGCAGGTAATGTAGCTTTCTTAATCAAACCCCCCCCAACCCCCCATTAAACTCCGCAGATGTACATTCAACACAATCTTGCCAAACCCCGAAAACAAGACTAAATAATGCACAACACTTCACGAAGCCTAACTCTCGCACATCAACCATGTCAACTCTATTCACCCAACAAATCCAACAGAACTTCCTCTTTTCTTTCCCCCCCCCTTTTAATACCAACATGCTACTTTAATCAATAAAATTTCCATAGACAGGTATCCCCCTAGATCTGATTTTCCAAACCTGCAAACCCCTCTTCCCCC

Sample no. ZDT4

gttaatgtagcttaataatataaagcaaggcactgaaaatgcctagatgagtattcctactccataaacacataggcttggtcctagcctttttattagttattaatagaattacacatgcaagtatccgcgccccagtgagaatgccctctaaatcgcactctacgatcaaaaggagcaggtatcaagcacactagaaagtagcttataacaccttgctcagccacacccccacgggacacagcagtgataaaaattaagccatgaacgaaagttcgactaagttatattaaattagggttggtaaaattcgtgccagccaccgcggtcatacgattaacccaaattaataaaatcccggcgtaaagcgtgtcaaagaccttacaccaaaataaagttaaaacccagttaagctgtaaaaagctacaaccaaagtaaaatagactacgaaagtgactttaatacctctgaccacacgatagctaagacccaaactgggattagataccccactatgcttagccctaaactaaaatagctcatcacaacaaagttattcgccagagtactactagcaacagcctaaaactcaaaggacttggcggtgctttacatccctctagaggagcctgttccataatcgataaaccccgataaaccccaccatcccttgctaattcagcctatataccgccatcttcagcaaaccctaaacaaggcaccgaagtaagcacaatcatccaacataaaaacgttaggtcaaggtgtagcccatggggtggagagaaatgggctacattttctactctaagaacaaaaacttaacccaaacgaaagtctctatgaaattggagaccgaaggaggatttagcagtaaattaagaatagagagcttaattgaatcaggccatgaagcgcgcacacaccgcccgtcaccctccttaaatatcacaaaccacaattttaacacaaaaccgtggcccaaacatatgaaaggagacaagtcgtaacaaggtaagtataccggaaggtgtacttggataaccaaagtgtagcttaaacaaagcatccagcttacacctagaagatttcactcagaatgaacactttgaactaaagctagcccaaacgacatccaactcaactacccttagtcacttaactaaaacattcactaaaccattaaagtataggagatagaaattttaacttggcgctatagagaaagtaccgtaagggaacgatgaaagatacattaaaagtaccaaacagcaaagcttaccccttttaccttttgcataatgatttaactagaataaacttagcaaagagaacttaagctaagcaccccgaaaccagacgagctacctacgaacagttacaaagaaccaactcatctatgtcgcaaaatagtgagaagattcataggtagaggtgaaaagcccaacgagcctggtgatagctggttgtccagaaacagaatctcagttcaaatttaagtttacctaaaaaccacccaattctaatgtaaacttaaattataatctaaaaaggtacagctttttagatacaggatacaacctttattagagagtaagaataagataatcccatagttggcttaaaagcagccatcaattaagaaagcgttcaagctcaacgtcacatttatcttaatcccaaaaataaactcaaactaactcctaatcttatactggactattc-tatcaacacatagaagcaataatgttaatatgagtaacaagaattatttctccttgcataagcctatatcagaacgaatactcactgatagttaacaacaaaatagacacaacccaaaaactaaccacctatttaaattattgttaacccaacacaggcatgcgcccataaggaaagattaaaagaagtaaaaggaactcggcaaacacaaaccccgcctgtttaccaaaaacatcacctctagcatttccagtattagaggcactgcctgcccagtgacatctgtttaaacggccgcggtatcctaaccgtgcaaaggtagcataatcacttgttctctaaatagggacttgtatgaatggccacacgagggttttactgtctcttacttccaatcagtgaaattgaccttctcgtgaagaggcgagaataaccaaataagacgagaagaccctatggagctttaattaactgattcacaaaaaacaacatacaaacctaaccctcagggacaacaaaacttttgattgaatcagcaatttcggttggggtgacctcggagaacaaaacaacctccgagtgatttaaacctagactaaccagtcaaaatacataatcacttattgatccaaaccattgatcaacggaacaagttaccctagggataacagcgcaatcctattccagagtccatatcgacaattagggtttacgacctcgatgttggatcaagacatcctaatggtgcaaccgctattaagggttcgtttgttcaacgattaaagtcttacgtgatctgagttcagaccggagtaatccaggtcggtttctatctattctacacttttcccagtacgaaaggacaagaaaagtagggcccactttacaagaagcgcccttaaactaatagatgacataatctaaatctaactaatttataaacctcaccgccctagaacagggctcgttagggtggcagagcccggaaattgcataaaacttaaacctttatacccagaggttcaattcctctccctaacaacatgttcataattaacgttctcctcctaattgtcccaatcctgctcgccgtagcattcctcacactagttgaacgaaaagtcttaggctacatacaacttcgcaaaggacccaacatcgtaggcccctatggcctactacaacccattgccgatgccctcaaactatttatcaaagaaccactacaaccactaacatcatcgacatccatatttatcatcgcaccaatcctagccctcaccctagccttaactatatgaatccctctccccataccgtacccactaattaacataaacctaggaattctattcatattagccatatccagcctagctgtctactcaatcctttgatcaggatgagcctcaaactcaaaatacgctctaatcggagccctacgagcagtagcacaaaccatctcatatgaagtaactctagcaatcattctactctcagtcctcctaataagcggatcattcacactatcaacacttatcactacccaagaatacttatgattaatcttcccatcatgacccttagccataatatgatttatctcaacattagccgaaaccaaccgagctccatttgacctaacagaaggagaatcagaactcgtctccggattcaacgttgaatacgcagccggcccattcgctctattcttcctagcagaatatgcaaacatcatcataatgaacatcttcacaacaactctatttctaggagcatttcacaacccctacctaccagaactctactcaattaattttaccatcaaagccctccttctaacatgttccttcctatgaatccgagcatcctacccgcgattccgatatgaccaacttatacacctcctatgaaaaaactttctaccactcacactagccctctgcatatgacatgtctcgctaccaatcatactatccagcatcccaccacaaacataagaaatatgtctgacaaaagagttactttgatagagtaaaacatagaggttcaaaccctcttatttctagaaccacaggaatcgaacctgctcctgagaattcaaaatcctccgtgctaccaaattacaccatgccctacaagtaaggtcagctaaataagctatcgggcccataccccgaaaatgttggattacacccttcccgtactaataaacccccttatcctcacaattatcctaataacagtttttctaggaactataatcgtcataataagctcacactgactaataatctgaatcggattcgaaataaatctactagccattatccccatcctaataaaaaaatacagcccccgagccatagaagcctccaccaaatacttcctaacccaagccaccgcatctatactcctcataatagcaattatcatcaacctgatgcactcaggccaatgaacaatcacaaaaattttcaaccccacagcatccatcatcataacttcagcccttgccataaaacttggactcacaccattccacttctgagtgcccgaagtcacacagggcatctcactaacatcaggcctcattctactcacatgacaaaaactagccccaatatcaatcctatatcaaatctcaccctcaatcaacctgaatatcttacttaccatagccgtactgtcaatcctagtaggaggctgaggtggtctcaaccaaacccaactacgaaaaattatagcatactcgtcgatcgcacatataggatgaataatggctgtcctggcatacaacccaacactgacaatactaaacatactaatttacattgtaataacactcacaatattcatactgtttatccacagctcctccactacaacactatcactctcacacacatgaaacaaaacacctctaaccaccacactaattctaatcaccttgctatctataggaggcctccccccactatcaggattcatacccaaatgaataatcatccaagaactcaccaaaaacagcagcatcattctccccactctaatggctatcatagcactactcaatctctacttctacatacgactaacctattccacctccctaaccatattcccatccacaaacaacataaaaataaaatgacaattcgaagccaaacaaattaccctcctacccccattaatcgtcgcatcttccctactcctccccctaacccccatactatcaattctggactaggaatttaggttagcatccagaccaagagccttcaaagctctaagcaagtatattcacttaattcctgcacactaaggactgcaagactctatctcacatcaattgaacgcaaatcaaacactttcattaagctaagcccttactagattggtgggctatcatcccacgaaactttagttaacagctaaacaccctaatcaactggcttcaatctacttctcccgccgcctaaaaaaaaaggcgggagaagccccggcagaattaaagctgctccttcgaatttgcaattcaatgtgaaattcaccacgggacttgataagaagaggattccaacccctgtctttagatttacagtctaatgcttactcagccatcttacctatgttcatcaaccgctgactattttcgactaaccacaaagacatcggcactctgtacctcctattcggcgcttgagctggaatagtaggaaccgccctaagcctcctaatccgtgctgaattaggccaacctgggaccctactaggagatgaccagatctacaatgttattgtaaccgcccacgcattcgtaataattttctttatagtcatacccattatgatcggaggatttgggaactgattagtccccttaataattggagcacctgacatagctttcccccgaataaacaacataagcttctgattacttcccccatcattcctgctccttcttgcttcctcaataattgaagcaggcgctggaacaggctgaaccgtataccctcccctagctgggaatctagcgcatgcaggggcctctgttgacttaaccattttctctctccacctagctggtgtatcttcaatcctaggtgccatcaatttcattactacaatcatcaacataaaaccgccagccctatcccaatatcaaactcctctattcgtttgatccgtccttattacggcagtactccttcttctagcccttccagtcctagcagcaggcattaccatgcttctcacagaccgtaacctaaacaccaccttcttcgaccctgcaggaggaggagacccaatcctttaccaacacctattctgattcttcggacaccccgaagtctacattctcatcctgccaggttttggtataatctcacacatcgttacatattattcaggtaaaaaggaaccttttggctacatgggtatagtgtgagctataatatccattggctttctaggcttcatcgtatgagctcaccacatgtttacagtaggtatagacgtcgatacacgagcatacttcacatcagctaccataatcatcgccatccctactggtgtaaaagtattcagctgactagccaccctacacggaggaaatatcaaatgatccccggctatactctgagctctaggcttcatcttcttattcacagtaggaggcctaacaggtatcgtcttggctaattcatccctagatattgttctccatgatacttattatgtagtagcacatttccactatgtcctgtccataggagcagtcttcgccattataggaggatttgttcactgattccctctattctcaggatatacactcaaccaaacctgagcaaaaatccactttacaatcatattcgtaggggtcaatataactttcttcccacaacacttccttggcctctcaggaataccacgacgctactctgattacccagacgcatacacaacatgaaacaccatctcatccataggatctttcatctcactcacagcagtaatactaataatcttcatgatttgggaagcattcgcatctaaacgagaagtgtctacagtagaattaacctcaactaacctagaatgactacacggatgccccccaccataccacacattcgaagaacccgcttatgtaaacctaaaataagaaaggaaggaatcgaaccccctctaactggtttcaagccaatatcataaccactatgtctttctccattaaacgaggtattagtaaaaattacataactttgtcaaagttaaattataggttgaacccctatatacctctatggcctaccccttccaattaggattccaagacgcaacatcccccattatagaagaactcctacacttccatgaccacacgctaataatcgtattcctaattagctctctagtattatacattatctcatcaatactaacaactaaattaacccacactagcaccatagacgcccaagaagtagaaacaatttgaacaattctaccagccattatccttattctaattgccctcccatccctgcgaattctatatataatagatgaaatcaacaacccatctctcacggtcaaaacaatgggccaccaatgatactgaagttacgaatataccgattacgaggacctgaccttcgattcctacatgatccccacatcagacctaaaaccaggagaattacgtcttctagaagtcgacaaccgagtggttctccctatggaaataactatccgaatactaatctcatccgaagacgtcctacactcatgagctgtcccctccctaggcctaaaaacagacgccatccctgggcgcctaaatcaaacaactctcgtagcctcccgaccaggtctttactacggccaatgttcggagatctgcggatcaaaccacagcttcataccaattgtccttgaactagtcccactgaaacacttcgaagaatgatctgcatcaatactataaaatcactaagaagctattatagcgttaaccttttaagttagagactgagggttcaaccccctccctagtgatatgccacagttggatacatcaacatgatttattaatatcgtctcaatgatcttaactctatttattgtatttcaactaaaaatctcaaagcactcttacccaacacacccagaagcaaaaacaactaaaataaccaaacacttcaccccttgagaatcaaaatgaacgaaaatctattcgcctctttcgctaccccaacagtaataggcctccctattgtgattctaatcatcatattccccagcatcctattcccttcatccaaccgactaatcaacaaccgcctaatctcaattcaacaatgactagtccaacttacatcaaaacaaataatagctattcacaacaacaaaggacaaacctgaaccctcatactcatgtcactaatcctattcattggctcgacaaacttattaggcctactacctcactcatttacaccaacaacacaactatcaataaatctaggcatagccattcccctgtgagcaggaacagtattcataggcttccgtcataaaacaaaagcagccctagctcactttctgcctcaaggaacacccattttcctcattcccatgctagtaattattgagactatcagcctatttattcaacctgtagctctagccgtacggctaactgctaacattactgccggacatcttctaattcaccttatcggaggagcaacactagccctcataaatattagcccctcaacagcccttattacatttattatcttaattctgctgactatcctcgaatttgcagtagccataatccaagcctacgtattcactcttctagtaagcctctacctacatgataacacctaatgacccaccaaacccacgcctaccacatagtaaatcccagcccatgaccacttacaggagccctatcagccctcctaataacatcaggattagccatgtgattccactttaactcaactctacttctagctttaggactattaaccaacatcctcaccatatatcaatgatgacgagacatcatccgagaaagcacatttcaaggccaccacacattaatcgtccaaaaaggactccgatacggcataatccttttcatcatctcagaagtcttcttcttctctggcttcttttgagccttctaccactcaagcctagctcccacacccgaactaggcggctgctgaccacccacaggtatccaccccttaaaccccctagaagtccccttactcaacacctcagtgctcctagcatctggggtctctatcacctgagcccaccatagcctgatagaagggaaccgtaaaaacatgctccagggcctattcatcacaatctcactaggcgtatacttcaccctcctccaagcctcagaatactacgaagcctcatttaccatttcagatggggtgtatggatcgacatttttcgtggcaacaggattccacggactacacgtaatcatcgggtccaccttccttatcgtatgcttcctacgccaactaaaattccactttacatccagccaccatttcggattcgaagcagccgcttgatactgacacttcgtcgacgtagtttgactattcttatatgtctctatctattgatgaggatcctattcttttagtattgaccagtacaattgacttccaatcaatcagcttcggtacaacccgaaaaagaataataaacctcatactaacactcctcaccaacacattgctagcctcactactcgtactcatcgcattctgactaccgcaactaaatatctatgcagaaaaaaccagcccatacgaatgcggatttgacccaatagggtcagcacgcctccctttctcaataaaatttttcttagtggccattacatttctgctgttcgacctagaaatcgccctcctgctaccccttccatgagcatcccagacaactaacctgaacaccatactcatcatagcactaatcctaatctctctcctagctatcagcctggcctatgaatgaacccaaaaaggactagaatgaactgagtatggtaattagtttaaactaaaacaaatgatttcggctcattaaactatgattaacttcataattaccaacatgtcactagtccacattaatatcttcctagcattcacagtctccctcgtaggcctactaatgtaccgatcccacctaatatcctcactcctatgcctagaagggataatactgtcactattcgtcatagcaaccataatagtcctaaacacccatttcacactagccagcataatacccattatcctactagtattcgctgcctgcgaagcagcattagggctatccttactagtcatagtctccaatacttatggagtggaccacgtacaaaaccttaacctcctccaatgctaaaaatcatcattcccacaatcatactaatacccctcacatgactatcaaaaaagaacataatctgaattaacaccacaacctacagtctattaatcagcctcatcagcctatccctcctaaatcagcctagcaacaatagcctaaacttctcactaatattcttctctgaccccctatcagctccactactagtactaacaacatgactactaccactaatactcatagccagccaacatcacctatccaaagaaccactaatccgaaaaaaactctacatcaccatgctaaccatacttcaaactttcctaattataacttttaccgccacagaactaatctctttctacatcttattcgaagccacattagttccaacactaattatcatcacccgctgaggcaaccaaacagaacgcctaaacgcaggcctctactttctattctacacactaataggctctctcccacttttagtcgcactaatctccatccaaaacctaacaggctcactaaacttcctactaatccaatactgaaaccaaacactacccgactcctgatctaatgtcttcctatgattagcatgtataatagcatttatagttaaaatgcccctatatggtctccacctctgacttccaaaagcccatgtagaagccccaatcgctggatccatagtactagcagccatcctactaaaactaggaggctacggaatactgcgaattacaacaatactaaaccctcaaaccaactttatagcctaccctttccttatactatctctatgaggaataatcataactagctccatctgtttacgacaaactgatctaaaatcactcattgcatactcctctgtcagtcatatagccctagtaattgtagccgtccttatccaaacaccatgaagctatatgggggccacagccctaatgattgcccacggtcttacgtcatcaatactattctgcctagcaaactcaaactacgaacgcactcacagccgaaccataatcctagcccgcggacttcaaacactcctccccctcatagcagcctgatgactattagccagcttaaccaacctggccctccctcccagcattaacctaatcggagaactattcgtagtaatatcgtcattctcatgatcaaatattactatcatcctcatgggaactaacatcaccatcaccgctctctactccctctatatactaatcaccacacaacgaggaaaatacacacaccacatcaataacatcaaaccctcatttacacgagaaaacgcactcatagccctccacataactcccctactactcctatcacttaaccctaaaatcatcctaggctttacgtactgtaaatatagtttaacaaaaacactagattgtggatctagaaatagaaactcaacatttcttatttaccgagagagtatgcaagaactgctaattcatgcctccatgtctgacaaacatggctctctcccaaacttttaaaggatgggagctatccgttggtcttaggaaccaaaaaattggtgcaactccaaataaaagtaatcaacatattctcctccctcatactagtttcactatcagtattaaccctgcctatcatatcatcaatcctcaatacccacaaaaacaacacgtacccacatcacgtaaaaaatattatctcatatgccttcattaccagcctaattcccaccataatattcatccactctggacaagaaacaatcatctcaaactgacactgagtaactatacaaaccctcaaactatctctaagcttcaaactagactacttctcaatagtcttcgtaccggtagccctattcgtaacatgatctatcatagaattctctttatgatacatgcactcggatccttacatcacccgattttttaaatacctactcacattcctcattaccataataatcctagtcacagccaataacctcttccaactgttcattggatgagaaggagtaggcatcatatcattcttactaatcggatgatggtatggtcgaacagacgccaacactgcggccctccaagcaatcctatataaccgcatcggagatattggcttcatcatagccatggcctgattcctattcaacaccaacacatgagacctacaacaaatcttcatacttgaccccaacctcaccaacctcccactcctaggccttctcctagccgcaactggtaaatctgcccaattcggactccacccatgacttccctcagccatagaaggccccacaccagtctcagccctactccactccagcacaatagttgtagcaggcgtcttcctactaatccgtttccacccactaatagaaaacaacaaaaccatccagtcactcaccctatgcctaggagctatcaccacactattcacagcaatctgtgcactcacccaaaacgacatcaaaaaaatcatcgccttctccacctccagccaactaggtctgataatcgtaaccatcggcattaatcagccctacctagcattccttcacatctgcactcacgcattcttcaaagccatactattcatatgctccggatccattatccacagcctaaatgacgagcaggacatccgaaaaataggcgggctgtttaatgcaatacctttcaccaccacatccctaattattggcagcctcgcactcaccggcatacctttcctcacaggcttttactccaaagacctcatcatcgaaaccgccaacacatcgtacaccaacgcctgagccctgctaataacactcatcgccacatccctcacagctgtctacagcacccgaatcatcttcttcgcactcctaggacaacctcgcttcctccccttaacttcaatcaacgaaaataatcctttcctaattaactccattaaacgcctcttaattggcagcattttcgccggattctttatctctaacaatatctacccctcaaccgtcccagaaataaccatacctacctacataaaacttaccgccctcgcagtaaccatcctagggtttacactagccctagaactaagcttaattacgcataatctaaaactaggacaccccaccaacatatttaaattctccaacctcctaggttactacccaacaatcatacatcgactcccaccgctcgcaaacctatcaataagccaaaaatcagcatcacttctactagactcaatctgactagaaagcatcctaccaaaatccatctcccaattccagataaaaacctcaatcctaatttccacccaaaaaggccaaatcaagctatatttcctctcattccttatcaccctcaccctaagcatactactctttaatctccacgagtaacctctaaaattaccaaaactccaacaagcaacgaccaaccagtcacaatcacaacccaagccccataactatacaatgcagcaacccccataatctcctcactaaacaccccagaatctccagtatcatagatagctcaatcccctacaccactaaacttaaacactacccccacttcctcactcttcagaacatataaaaccagcataacctctatcaacaaccccaaaagaaacacccccataacagtcgtattagacacccacacctcagggtattgctcagtagccatagccgttgtataaccaaaaacaaccaacattcctcccaaataaatcaaaaacaccatcaaccctaaaaaggaccctccaaaattcataatgatgccacaaccaacccctccacttacaatcaacactaaacccccataaataggcgaaggttttgaagaaaaacctacaaaaccaataacaaagataacgctcaaaataaacacaatatatgtcatcattattcctacgtggaatctaaccacgactaatgacatgaaaaatcatcgttgtatttcaactataagaacactaatgacaaacatccgaaaatctcacccgctaattaaaatcatcaatcactccttcatcgacctaccagccccctcaaacatttcatcatgatgaaactttggctccctcctaggaatctgcctaatcctccaaatccttacaggtctattcctagccatacactacacatcagacacaacaaccgccttctcatccgttactcacatctgccgagatgtcaactacggatgaatcattcgctacctccacgctaacggagcatccatatttttcatctgcctcttcatccacgtaggacgcggcctctactatggctcctacacattcctagaaacatgaaacattggaattatcctacttttcacagtaatagccacagcattcataggctatgtcctaccatgaggccaaatatccttctgaggagcaacagtcatcacaaacctcctatcagcaatcccctacatcggcactaccctcgtcgaatgaatctgaggtggattctcagtagacaaagccacccttacccgattttttgccttccactttattctaccctttatcatcacagccctggtagtcgtccatctactattcctccacgaaacaggatccaacaacccctcaggaatcccatctgacatagacaaaatcccattccacccgtactacacaattaaagacatcctaggactcctcctcctaatcctactcctactgaccctagtactattctcccctgacctcctaggagacccggacaactacaccccagctaaccctcttagcactccccctcatattaagccagagtgatacttcctatttgcctacgccatcctacgctctattcccaacaaactaggcggcgtattagcccttatcctttccatcttaatcttagcactcatccccactctacacatatcaaaacaacgaagcataatattccgacctcttagtcaatgcgtgttctgactcttagtagcagacttactaacactaacatgaatcggcggccagccagtggaacacccatacgtaattatcggccaactggcctcaatcctctacttctccctaattctcatcttcataccactcgcaagcaccatcgaaaacaatcttctaaagtgaagagtccctgtagtatatcacatattactctggtcttgtaaaccagaaaagggggaaacattccccccaaggactgtcaaggaagaagccctagctccaccatcagcacccaaagctgaaattctacttaaactattccttgaatttcctcccctaaacgacaacaattcatcctcgtgtgctatgtcagtattaaaacataccctacgcaatatcgtacataacccaacacacaatatcctattaacgccctatgtacgtcgtgcattaggttgtttgccccatgaataataagcatgtacataatattatttatcttacatgagtacatcatattattaatcgtgcataccccattcaagtcaaatcatttccagtcaacacgcatatcaccacccatattccacgagctcaatcaccaagccgcgggaaatcagcaacccttccaattacgtgtcccaatcctcgctccgggcccatccgagtgtgggggtttctacagtgaaactatacctggcatctggttctttcttcaggaccatctcacccaacctcgcccattctttccccttaaataagacatctcgatggactaatgactaatcagcccatgctcacacataactgtggtttcatgcatttggtatcttttttattttggggatgctatgactcagctatggccgtcaaaggccccgacgcagtcaattaaattgaagctggacttaaattgaacgttattactccgcatacaacaaccataaggtgttattcagtccatggtagcaggacatagagaataannnnnnnnnacacacaccaagcaggtaatgtaactttcttaatcaaaccccccccaaccccccattaaactccgcagatgtacattcaacacaatcttgccaaaccccgaaaacaagactaaataatgcacaacacttcacgaagcctaactctcgcacatcaaccatgtcaactctattcactcaacaaatccaacagaacttcctcttttctttcccccccccttttaataccaacatgctactttaatcaataaaatttccatagacaggtatccccctagatctgattttccaaacctgcaaacccctcttccccc

Sample no. ZDT9

gttaatgtagcttaataatataaagcaaggcactgaaaatgcctagatgagtattcctactccataaacacataggcttggtcctagcctttttattagttattaatagaattacacatgcaagtatccgcgccccagtgagaatgccctctaaatcgcactctacgatcaaaaggagcaggtatcaagcacactagaaagtagcttataacaccttgctcagccacacccccacgggacacagcagtgataaaaattaagccatgaacgaaagttcgactaagttatattaaattagggttggtaaaattcgtgccagccaccgcggtcatacgattaacccaaattaataaaatcccggcgtaaagcgtgtcaaagaccttacaccaaaataaagttaaaacccagttaagctgtaaaaagctacaaccaaagtaaaatagactacgaaagtgactttaatacctctgaccacacgatagctaagacccaaactgggattagataccccactatgcttagccctaaactaaaatagctcatcacaacaaagttattcgccagagtactactagcaacagcctaaaactcaaaggacttggcggtgctttacatccctctagaggagcctgttccataatcgataaaccccgataaaccccaccatcccttgctaattcagcctatataccgccatcttcagcaaaccctaaacaaggcaccgaagtaagcacaatcatccaacataaaaacgttaggtcaaggtgtagcccatggggtggagagaaatgggctacattttctactctaagaacaaaaacttaacccaaacgaaagtctctatgaaattggagaccgaaggaggatttagcagtaaattaagaatagagagcttaattgaatcaggccatgaagcgcgcacacaccgcccgtcaccctccttaaatatcacaaaccacaattttaacacaaaaccgtggcccaaacatatgaaaggagacaagtcgtaacaaggtaagtataccggaaggtgtacttggataaccaaagtgtagcttaaacaaagcatccagcttacacctagaagatttcactcagaatgaacactttgaactaaagctagcccaaacgacatccaactcaactacccttagtcacttaactaaaacattcactaaaccattaaagtataggagatagaaattttaacttggcgctatagagaaagtaccgtaagggaacgatgaaagatacattaaaagtaccaaacagcaaagcttaccccttttaccttttgcataatgatttaactagaataaacttagcaaagagaacttaagctaagcaccccgaaaccagacgagctacctacgaacagttacaaagaaccaactcatctatgtcgcaaaatagtgagaagattcataggtagaggtgaaaagcccaacgagcctggtgatagctggttgtccagaaacagaatctcagttcaaatttaagtttacctaaaaaccacccaattctaatgtaaacttaaattataatctaaaaaggtacagctttttagatacaggatacaacctttattagagagtaagaataagataatcccatagttggcttaaaagcagccatcaattaagaaagcgttcaagctcaacgtcacatttatcttaatcccaaaaataaactcaaactaactcctaatcttatactggactattc-tatcaacacatagaagcaataatgttaatatgagtaacaagaattatttctccttgcataagcctatatcagaacgaatactcactgatagttaacaacaaaatagacacaacccaaaaactaaccacctatttaaattattgttaacccaacacaggcatgcgcccataaggaaagattaaaagaagtaaaaggaactcggcaaacacaaaccccgcctgtttaccaaaaacatcacctctagcatttccagtattagaggcactgcctgcccagtgacatctgtttaaacggccgcggtatcctaaccgtgcaaaggtagcataatcacttgttctctaaatagggacttgtatgaatggccacacgagggttttactgtctcttacttccaatcagtgaaattgaccttctcgtgaagaggcgagaataaccaaataagacgagaagaccctatggagctttaattaactgattcacaaaaaacaacatacaaacctaaccctcagggacaacaaaacttttgattgaatcagcaatttcggttggggtgacctcggagaacaaaacaacctccgagtgatttaaacctagactaaccagtcaaaatacataatcacttattgatccaaaccattgatcaacggaacaagttaccctagggataacagcgcaatcctattccagagtccatatcgacaattagggtttacgacctcgatgttggatcaagacatcctaatggtgcaaccgctattaagggttcgtttgttcaacgattaaagtcttacgtgatctgagttcagaccggagtaatccaggtcggtttctatctattctacacttttcccagtacgaaaggacaagaaaagtagggcccactttacaagaagcgcccttaaactaatagatgacataatctaaatctaactaatttataaacctcaccgccctagaacagggctcgttagggtggcagagcccggaaattgcataaaacttaaacctttatacccagaggttcaattcctctccctaacaacatgttcataattaacgttctcctcctaattgtcccaatcctgctcgccgtagcattcctcacactagttgaacgaaaagtcttaggctacatacaacttcgcaaaggacccaacatcgtaggcccctatggcctactacaacccattgccgatgccctcaaactatttatcaaagaaccactacaaccactaacatcatcgacatccatatttatcatcgcaccaatcctagccctcaccctagccttaactatatgaatccctctccccataccgtacccactaattaacataaacctaggaattctattcatattagccatatccagcctagctgtctactcaatcctttgatcaggatgagcctcaaactcaaaatacgctctaatcggagccctacgagcagtagcacaaaccatctcatatgaagtaactctagcaatcattctactctcagtcctcctaataagcggatcattcacactatcaacacttatcactacccaagaatacttatgattaatcttcccatcatgacccttagccataatatgatttatctcaacattagccgaaaccaaccgagctccatttgacctaacagaaggagaatcagaactcgtctccggattcaacgttgaatacgcagccggcccattcgctctattcttcctagcagaatatgcaaacatcatcataatgaacatcttcacaacaactctatttctaggagcatttcacaacccctacctaccagaactctactcaattaattttaccatcaaagccctccttctaacatgttccttcctatgaatccgagcatcctacccgcgattccgatatgaccaacttatacacctcctatgaaaaaactttctaccactcacactagccctctgcatatgacatgtctcgctaccaatcatactatccagcatcccaccacaaacataagaaatatgtctgacaaaagagttactttgatagagtaaaacatagaggttcaaaccctcttatttctagaaccacaggaatcgaacctgctcctgagaattcaaaatcctccgtgctaccaaattacaccatgccctacaagtaaggtcagctaaataagctatcgggcccataccccgaaaatgttggattacacccttcccgtactaataaacccccttatcctcacaattatcctaataacagtttttctaggaactataatcgtcataataagctcacactgactaataatctgaatcggattcgaaataaatctactagccattatccccatcctaataaaaaaatacagcccccgagccatagaagcctccaccaaatacttcctaacccaagccaccgcatctatactcctcataatagcaattatcatcaacctgatgcactcaggccaatgaacaatcacaaaaattttcaaccccacagcatccatcatcataacttcagcccttgccataaaacttggactcacaccattccacttctgagtgcccgaagtcacacagggcatctcactaacatcaggcctcattctactcacatgacaaaaactagccccaatatcaatcctatatcaaatctcaccctcaatcaacctgaatatcttacttaccatagccgtactgtcaatcctagtaggaggctgaggtggtctcaaccaaacccaactacgaaaaattatagcatactcgtcgatcgcacatataggatgaataatggctgtcctggcatacaacccaacactgacaatactaaacatactaatttacattgtaataacactcacaatattcatactgtttatccacagctcctccactacaacactatcactctcacacacatgaaacaaaacacctctaaccaccacactaattctaatcaccttgctatctataggaggcctccccccactatcaggattcatacccaaatgaataatcatccaagaactcaccaaaaacagcagcatcattctccccactctaatggctatcatagcactactcaatctctacttctacatacgactaacctattccacctccctaaccatattcccatccacaaacaacataaaaataaaatgacaattcgaagccaaacaaattaccctcctacccccattaatcgtcgcatcttccctactcctccccctaacccccatactatcaattctggactaggaatttaggttagcatccagaccaagagccttcaaagctctaagcaagtatattcacttaattcctgcacactaaggactgcaagactctatctcacatcaattgaacgcaaatcaaacactttcattaagctaagcccttactagattggtgggctatcatcccacgaaactttagttaacagctaaacaccctaatcaactggcttcaacctacttctcccgccgcctaaaaaaaaaggcgggagaagccccggcagaattaaagctgctccttcgaatttgcaattcaatgtgaaattcaccacgggacttgataagaagaggattccaacccctgtctttagatttacagtctaatgcttactcagccatcttacctatgttcatcaaccgctgactattttcgactaaccacaaagacatcggcactctgtacctcctattcggcgcttgagctggaatagtaggaaccgccctaagcctcctaatccgtgctgaattaggccaacctgggaccctactaggagatgaccagatctacaatgttattgtaaccgcccacgcattcgtaataattttctttatagtcatacccattatgatcggaggatttgggaactgattagtccccttaataattggagcacctgacatagctttcccccgaataaacaacataagcttctgattacttcccccatcattcctgctccttcttgcttcctcaataattgaagcaggcgctggaacaggctgaaccgtataccctcccctagctgggaatctagcgcatgcaggggcctctgttgacttaaccattttctctctccacctagctggtgtatcttcaatcctaggtgccatcaatttcattactacaatcatcaacataaaaccgccagccctatcccaatatcaaactcctctattcgtttgatccgtccttattacggcagtactccttcttctagcccttccagtcctagcagcaggcattaccatgcttctcacagaccgtaacctaaacaccaccttcttcgaccctgcaggaggaggagacccaatcctttaccaacacctattctgattcttcggacaccccgaagtctacattctcatcctgccaggttttggtataatctcacacatcgttacatattattcaggtaaaaaggaaccttttggctacatgggtatagtgtgagctataatatccattggctttctaggcttcatcgtatgagctcaccacatgtttacagtaggtatagacgtcgatacacgagcatacttcacatcagctaccataatcatcgccatccctactggtgtaaaagtattcagctgactagccaccctacacggaggaaatatcaaatgatccccggctatactctgagctctaggcttcatcttcttattcacagtaggaggcctaacaggtatcgtcttggctaattcatccctagatattgttctccatgatacttattatgtagtagcacatttccactatgtcctgtccataggagcagtcttcgccattataggaggatttgttcactgattccctctattctcaggatatacactcaaccaaacctgagcaaaaatccactttacaatcatattcgtaggggtcaatataactttcttcccacaacacttccttggcctctcaggaataccacgacgctactctgattacccagacgcatacacaacatgaaacaccatctcatccataggatctttcatctcactcacagcagtaatactaataatcttcatgatttgggaagcattcgcatctaaacgagaagtgtctacagtagaattaacctcaactaacctagaatgactacacggatgccccccaccataccacacattcgaagaacccgcttatgtaaacctaaaataagaaaggaaggaatcgaaccccctctaactggtttcaagccaatatcataaccactatgtctttctccattaaacgaggtattagtaaaaattacataactttgtcaaagttaaattataggttgaacccctatatacctctatggcctaccccttccaattaggattccaagacgcaacatcccccattatagaagaactcctacacttccatgaccacacgctaataatcgtattcctaattagctctctagtattatacattatctcatcaatactaacaactaaattaacccacactagcaccatagacgcccaagaagtagaaacaatttgaacaattctaccagccattatccttattctaattgccctcccatccctgcgaattctatacataatagatgaaatcaacaacccatctctcacggtcaaaacaatgggccaccaatgatactgaagttacgaatataccgattacgaggacctgaccttcgattcctacatgatccccacatcagacctaaaaccaggagaattacgtcttctagaagtcgacaaccgagtggttctccctatggaaataactatccgaatactaatctcatccgaagacgtcctacactcatgagctgtcccctccctaggcctaaaaacagacgccatccctgggcgcctaaatcaaacaactctcgtagcctcccgaccaggtctttactacggccaatgttcggagatctgcggatcaaaccacagcttcataccaattgtccttgaactagtcccactgaaacacttcgaagaatgatctgcatcaatactataaaatcactaagaagctattatagcgttaaccttttaagttagagactgagggttcaaccccctccctagtgatatgccacagttggatacatcaacatgatttattaatatcgtctcaatgatcttaactctatttattgtatttcaactaaaaatctcaaagcactcttacccaacacacccagaagcaaaaacaactaaaataaccaaacacttcaccccttgagaatcaaaatgaacgaaaatctattcgcctctttcgctaccccaacagtaataggcctccctattgtgattctaatcatcatattccccagcatcctattcccttcatccaaccgactaatcaacaaccgcctaatctcaattcaacaatgactagtccaacttacatcaaaacaaataatagctattcacaacaacaaaggacaaacctgaaccctcatactcatgtcactaatcctattcattggctcgacaaacttattaggcctactacctcactcatttacaccaacaacacaactatcaataaatctaggcatagccattcccctgtgagcaggaacagtattcataggcttccgtcataaaacaaaagcagccctagctcactttctgcctcaaggaacacccattttcctcattcccatgctagtaattattgagactatcagcctatttattcaacctgtagctctagccgtacggctaactgctaacattactgccggacatcttctaattcaccttatcggaggagcaacactagccctcataaatattagcccctcaacagcccttattacatttattatcttaattctgctgactatcctcgaatttgcagtagccataatccaagcctacgtattcactcttctagtaagcctctacctacatgataacacctaatgacccaccaaacccacgcctaccacatagtaaatcccagcccatgaccacttacaggagccctatcggccctcctaataacatcaggattagccatgtgattccactttaactcaactctacttctagctttaggactattaaccaacatcctcaccatatatcaatgatgacgagacatcatccgagaaagcacatttcaaggccaccacacattaatcgtccaaaaaggactccgatacggcataatccttttcatcatctcagaagtcttcttcttctctggcttcttttgagccttctaccactcaagcctagctcccacacccgaactaggcggctgctgaccacccacaggtatccaccccctaaaccccctagaagtccccttactcaacacctcagtgctcctagcatctggggtctctatcacctgagcccaccatagcctgatagaagggaaccgtaaaaacatgctccagggcctattcatcacaatctcactaggcgtatacttcaccctcctccaagcctcagaatactacgaagcctcatttaccatttcagatggggtgtatggatcgacatttttcgtggcaacaggattccacggactacacgtaatcatcgggtccaccttccttatcgtatgcttcctacgccaactaaaattccactttacatccagccaccatttcggattcgaagcagccgcttgatactgacacttcgtcgacgtagtttgactattcttatatgtctctatctattgatgaggatcctattcttttagtattgaccagtacaattgacttccaatcaatcagcttcggtacaacccgaaaaagaataataaacctcatactaacactcctcaccaacacattgctagcctcactactcgtactcatcgcattctgactaccgcaactaaatatctatgcagaaaaaaccagcccatacgaatgcggatttgacccaatagggtcagcacgcctccctttctcaataaaatttttcttagtggccattacatttctgctgttcgacctagaaatcgccctcctgctaccccttccatgagcatcccagacaactaacctgaacaccatactcatcatagcactaatcctaatctctctcctagctatcagcctggcctatgaatgaacccaaaaaggactagaatgaactgagtatggtaattagtttaaactaaaacaaatgatttcggctcattaaactatgattaacttcataattaccaacatgtcactagtccacattaatatcttcctagcattcacagtctccctcgtaggcctactaatgtaccgatcccacctaatatcctcactcctatgcctagaagggataatactgtcactattcgtcatagcaaccataatagtcctaaacacccatttcacactagccagcataatacccattatcctactagtattcgctgcctgcgaagcagcattagggctatccttactagtcatagtctccaatacttatggagtggaccacgtacaaaaccttaacctcctccaatgctaaaaatcatcattcccacaatcatactaatacccctcacatgactatcaaaaaagaacataatctgaattaacaccacaacctacagtctattaatcagcctcatcagcctatccctcctaaatcagcctagcaacaatagcctaaacttctcactaatattcttctctgaccccctatcagctccactactagtactaacaacatgactactaccactaatactcatagccagccaacatcacctatccaaagaaccactaatccgaaaaaaactctacatcaccatgctaaccatacttcaaactttcctaattataacttttaccgccacagaactaatctctttctacatcttattcgaagccacattagttccaacactaattatcatcacccgctgaggcaaccaaacagaacgcctaaacgcaggcctctactttctattctacacactaataggctctctcccacttttagtcgcactaatctccatccaaaacctaacaggctcactaaacttcctactaatccaatactgaaaccaaacactacccgactcctgatctaatgtcttcctatgattagcatgtataatagcatttatagttaaaatgcccctatatggtctccacctctgacttccaaaagcccatgtagaagccccaatcgctggatccatagtactagcagccatcctactaaaactaggaggctacggaatactgcgaattacaacaatactaaaccctcaaaccaactttatagcctaccctttccttatactatctctatgaggaataatcataactagctccatctgtttacgacaaactgatctaaaatcactcattgcatactcctctgtcagtcatatagccctagtaattgtagccgtccttatccaaacaccatgaagctatatgggggccacagccctaatgattgcccacggtcttacgtcatcaatactattctgcctagcaaactcaaactacgaacgcactcacagccgaaccataatcctagcccgcggacttcaaacactcctccccctcatagcagcctgatgactattagccagcttaaccaacctggccctccctcccagcattaacctaatcggagaactattcgtagtaatatcgtcattctcatgatcaaatattactatcatcctcatgggaactaacatcaccatcaccgctctctactccctctatatactaatcaccacacaacgaggaaaatacacacaccacatcaataacatcaaaccctcatttacacgagaaaacgcactcatagccctccacataactcccctactactcctatcacttaaccctaaaatcatcctaggctttacgtactgtaaatatagtttaacaaaaacactagattgtggatctagaaatagaaactcaacatttcttatttaccgagagagtatgcaagaactgctaattcatgcctccatgtctgacaaacatggctctctcccaaacttttaaaggatgggagctatccgttggtcttaggaaccaaaaaattggtgcaactccaaataaaagtaatcaacatattctcctccctcatactagtttcactatcagtattaaccctgcctatcatatcatcaatcctcaatatccacaaaaacaacacgtacccacatcacgtaaaaaatattatctcatatgccttcattaccagcctaattcccaccataatattcatccactctggacaagaaacaatcatctcaaactgacactgagtaactatacaaaccctcaaactatctctaagcttcaaactagactacttctcaatagtcttcgtaccggtagccctattcgtaacatgatctatcatagaattctctttatgatacatgcactcggatccttacatcacccgattttttaaatacctactcacattcctcattaccataataatcctagtcacagccaataacctcttccaactgttcattggatgagaaggagtaggcatcatatcattcttactaatcggatgatggtatggtcgaacagacgccaacactgcggccctccaagcaatcctatataaccgcatcggagatattggcttcatcatagccatggcctgattcctattcaacaccaacacatgagacctacaacaaatcttcatacttgaccccaacctcaccaacctcccactcctaggccttctcctagccgcaactggtaaatctgcccaattcggactccacccatgacttccctcagccatagaaggccccacaccagtctcagccctactccactccagcacaatagttgtagcaggcgtcttcctactaatccgtttccacccactaatagaaaacaacaaaaccatccagtcactcaccctatgcctaggagctatcaccacactattcacagcaatctgtgcactcacccaaaacgacatcaaaaaaatcatcgccttctccacctccagccaactaggtctgataatcgtaaccatcggcattaatcagccctacctagcattccttcacatctgcactcacgcattcttcaaagccatactattcatatgctccggatccattatccacagcctaaatgacgagcaggacatccgaaaaataggcgggctgtttaatgcaatacctttcaccaccacatccctaattattggcagcctcgcactcaccggcatacctttcctcacaggcttttactccaaagacctcatcatcgaaaccgccaacacatcgtacaccaacgcctgagccctgctaataacactcatcgccacatccctcacagctgtctacagcacccgaatcatcttcttcgcactcctaggacaacctcgcttcctccccttaacttcaatcaacgaaaataatcctttcctaattaactccattaaacgcctcttaattggcagcattttcgccggattctttatctctaacaatatctacccctcaaccgtcccagaaataaccatacctacctacataaaacttaccgccctcgcagtaaccatcctagggtttacactagccctagaactaagcttaattacgcataatctaaaactaggacaccccaccaacatatttaaattctccaacctcctaggttactacccaacaatcatacatcgactcccaccgctcgcaaacctatcaataagccaaaaatcagcatcacttctactagactcaatctgactagaaagcatcctaccaaaatccatctcccaattccagataaaaacctcaatcctaatttccacccaaaaaggccaaatcaagctatatttcctctcattccttatcaccctcaccctaagcatactactctttaatctccacgagtaacctctaaaattaccaaaactccaacaagcaacgaccaaccagtcacaatcacaacccaagccccataactatacaatgcagcaacccccataatctcctcactaaacaccccagaatctccagtatcatagatagctcaatcccctacaccactaaacttaaacactacccccacttcctcactcttcagaacatataaaaccagcataacctctatcaacaaccccaaaagaaacacccccataacagtcgtattagacacccacacctcagggtattgctcagtagccatagccgttgtataaccaaaaacaaccaacattcctcccaaataaatcaaaaacaccatcaaccctaaaaaggaccctccaaaattcataatgatgccacaaccaacccctccacttacaatcaacactaaacccccataaataggcgaaggttttgaagaaaaacctacaaaaccaataacaaagataacgctcaaaataaacacaatatatgtcatcattattcctacgtggaatctaaccacgactaatgacatgaaaaatcatcgttgtatttcaactataagaacactaatgacaaacatccgaaaatctcacccgctaattaaaatcatcaatcactccttcatcgacctaccagccccctcaaacatttcatcatgatgaaactttggctccctcctaggaatctgcctaatcctccaaatccttacaggtctattcctagccatacactacacatcagacacaacaaccgccttctcatccgttactcacatctgccgagatgtcaactacggatgaatcattcgctacctccacgctaacggagcatccatatttttcatctgcctcttcatccacgtaggacgcggcctctactatggctcctacacattcctagaaacatgaaacattggaattatcctacttttcacagtaatagccacagcattcataggctatgtcctaccatgaggccaaatatccttctgaggagcaacagtcatcacaaacctcctatcagcaatcccctacatcggcactaccctcgtcgaatgaatctgaggtggattctcagtagacaaagccacccttacccgattttttgccttccactttattctaccctttatcatcacagccctggtagtcgtccatctactattcctccacgaaacaggatccaacaacccctcaggaatcccatctgacatagacaaaatcccattccacccgtactacacaattaaagacatcctaggactcctcctcctaatcctactcctactgaccctagtactattctcccctgacctcctaggagacccggacaactacaccccagctaaccctcttagcactccccctcatattaagccagagtgatacttcctatttgcctacgccatcctacgctctattcccaacaaactaggcggcgtattagcccttatcctttccatcttaatcttagcactcatccccactctacacatatcaaaacaacgaagcataatattccgacctcttagtcaatgcgtgttctgactcttagtagcagacttactaacactaacatgaatcggcggccagccagtggaacacccatacgtaattatcggccaactggcctcaatcctctacttctccctaattctcatcttcataccactcgcaagcaccatcgaaaacaatcttctaaagtgaagagtccctgtagtatatcacatattactctggtcttgtaaaccagaaaagggggaaacattccccccaaggactgtcaaggaagaagccctagctccaccatcagcacccaaagctgaaattctacttaaactattccttgaatttcctcccctaaacgacaacaattcatcctcgtgtgctatgtcagtattaaaacataccctacgcaatatcgtacataacccaacnnnnnnnnnnnnnnnnnnnnnnnnnnnnnnnnnnnnnnnnnattgtttgccccatgaataataagcatgtacataatattatttatcttacatgagtacatcatattattaatcgtgcataccccattcaagtcaaatcatttccagtcaacacgcatatcaccacccatattccacgagctcaatcaccaagccgcgggaaatcagcaacccttccaattacgtgtcccaatcctcgctccgggcccatccgagtgtgggggtttctacagtgaaactatacctggcatctggttctttcttcaggaccatctcacccaacctcgcccattctttccccttaaataagacatctcgatggactaatgactaatcagcccatgctcacacataactgtggtttcatgcatttggtatcttttttattttggggatgctatgactcagctatggccgtcaaaggccccgacgcagtcaattaaattgaagctggacttaaattgaacgttattactccgcatacaacaaccataaggtgttattcagtccatggtagcaggacatagagaataagcacnnnnnncacacaccaagcaggtaatgtaactttcttaatcaaaccccccccaaccccccattaaactccgcagatgtacattcaacacaatcttgccaaaccccgaaaacaagactaaataatgcacaacacttcacgaagcctaactctcgcacatcaaccatgtcaactctatnnnnnnnnnnnnnnnnnnnnnnnnnnnnnnnttcttccccccccccttttaataccaacatgctactttaatcaataaaatttccatagacaggtatccccctagatctgattttccaaacctgcaaacccctnnnnnnnn
