# Supplementary material for: Molecular identification of late and terminal Pleistocene Equus ovodovi from northeastern China
Source: PLoS One. 2019 May 16;14(5):e0216883. doi: 10.1371/journal.pone.0216883 (PMC6522033; doi:10.1371/journal.pone.0216883)
Supplement: S1 Table — (DOCX) [file pone.0216883.s003.docx]

**S1 Table. Long range PCR primer pairs used in this study.**

| Primer pair | Primer sequence (5'->3') | Ta ℃ | Size (bp) |
| --- | --- | --- | --- |
| 1.6_Fwd | GCAGCCATCAATTAAGAAAGCG | 63 | 6191 |
| 7.8_Rev | CATGTTGATGTATCCAACTGTGGC |  |  |
| 6.3_Fwd | CACATCAGCTACCATAATCATCGC | 60 | 5024 |
| 11.3_Rev | GGTTAGGCTGGCTAATAGTCATC |  |  |
| Pr3_Fwd | CTCCCAAAAGCCCATGTAGA | 60 | 6853 |
| Pr3_Rev | GGCTACACCTTGACCTAACG |  |  |
| MAC_16.2_Fwd | ATTCCTCCGCATCAGCAACC | 63 | 2638 |
| MAC_2.0_Rev | TTTGCACGGTTAGGATACCG |  |  |
