# Supplementary material for: Molecular identification of late and terminal Pleistocene Equus ovodovi from northeastern China
Source: PLoS One. 2019 May 16;14(5):e0216883. doi: 10.1371/journal.pone.0216883 (PMC6522033; doi:10.1371/journal.pone.0216883)
Supplement: S2 Table — (DOCX) [file pone.0216883.s004.docx]

**S2 Table. Sequences used in this study to investigate the genetic diversity of the non-caballine equid members.**

| **Taxa** | **GenBank No.** |
| --- | --- |
| 1. *ovodovi* | JX312734, KY114520, ZDT4 (this study), ZDT7 (this study), ZDT9 (this study) |
| *E. burchellii* | JX312721, JX312729, JX312733, KM881680, NC_018781 |
| *E. grevyi* | JX312720, JX312722, JX312723, JX312725, NC_020432 |
| *E. zebra* | JX312717, JX312718, JX312719, JX312724, NC_018780, NC_020476 |
| *E. kiang* | JX312731, JX312732, NC_020433 |
| *E. hemionus* | HM118851, JX312728, JX312730, NC_016061, NC_018782 |
| *E. asinus* | KM881681, KT182635, KX669267, KX683425, MG885769, NC_001788, X97337 |
