# Supplementary material for: Molecular identification of late and terminal Pleistocene Equus ovodovi from northeastern China
Source: PLoS One. 2019 May 16;14(5):e0216883. doi: 10.1371/journal.pone.0216883 (PMC6522033; doi:10.1371/journal.pone.0216883)
Supplement: S3 Table — (DOCX) [file pone.0216883.s005.docx]

**S3 Table. Sequencing statistic results for NGS reads.**

| **Reference GenBank No.** | **Reference species** | **Sample** | **Total reads** | **Reads after trimming** | **Reads<30 bp** | **Total mapped reads** | **Unique mapped reads** | **Endogenous DNA** | **Duplication** | **Depth** | **Sequence length** |
| --- | --- | --- | --- | --- | --- | --- | --- | --- | --- | --- | --- |
|  |  | ZDT4 | 3587902 | 3148327 | 439575 | 2357128 | 16321 | 0.00518 | 0.99306 | 58.1 | 16435 |
| KY114520 | *E. ovodovi* | ZDT7 | 2794396 | 2352097 | 442299 | 1521966 | 30466 | 0.01295 | 0.91999 | 126.8 | 16440 |
|  |  | ZDT9 | 1528126 | 980967 | 547159 | 826789 | 20104 | 0.02049 | 0.97569 | 60.8 | 16358 |
|  |  | ZDT4 | 3587902 | 3148327 | 439575 | 1238795 | 9366 | 0.00297 | 0.99244 | 33.3 | 15563 |
| X97337 | *E. asinus* | ZDT7 | 2794396 | 2352097 | 442299 | 937534 | 20116 | 0.00855 | 0.97855 | 80.0 | 15708 |
|  |  | ZDT9 | 1528126 | 980967 | 547159 | 523683 | 13071 | 0.01332 | 0.97505 | 40.5 | 15255 |
|  |  | ZDT4 | 3587902 | 3148327 | 439575 | 1127956 | 7815 | 0.00248 | 0.99308 | 29.1 | 14400 |
| X79547 | *E. caballus* | ZDT7 | 2794396 | 2352097 | 442299 | 981101 | 17935 | 0.00762 | 0.98172 | 71.1 | 15147 |
|  |  | ZDT9 | 1528126 | 980967 | 547159 | 599575 | 12710 | 0.01295 | 0.97881 | 40.8 | 14295 |
