# Supplementary material for: Molecular identification of late and terminal Pleistocene Equus ovodovi from northeastern China
Source: PLoS One. 2019 May 16;14(5):e0216883. doi: 10.1371/journal.pone.0216883 (PMC6522033; doi:10.1371/journal.pone.0216883)
Supplement: S4 Table — (DOCX) [file pone.0216883.s006.docx]

**S4 Table. Number of nucleotide differences between *E. ovodovi* samples based on complete mitochondrial genomes.**

| Samples | JX312734 | KY114520 | ZDT7 | ZDT4 |
| --- | --- | --- | --- | --- |
| KY114520 | 120 |  |  |  |
| ZDT7 | 138 | 94 |  |  |
| ZDT4 | 138 | 95 | 67 |  |
| ZDT9 | 143 | 99 | 72 | 5 |
